# tRNA thiolation optimizes appressorium-mediated infection by enhancing codon-specific translation in *Magnaporthe oryzae*

**DOI:** 10.1093/nar/gkae1302

**Published:** 2025-01-07

**Authors:** Xinrong Zhang, Rongrong He, Yinan Li, Shuchao Ren, Shikun Xiang, Jing Zheng, Zhiguang Qu, Shu Zhou, Zhipeng Zhou, Xiao-Lin Chen

**Affiliations:** National Key Laboratory of Agricultural Microbiology, Huazhong Agricultural University, Wuhan 430070, China; Provincial Key Laboratory of Plant Pathology of Hubei Province, College of Plant Science and Technology, Huazhong Agricultural University, Wuhan 430070, China; National Key Laboratory of Agricultural Microbiology, Huazhong Agricultural University, Wuhan 430070, China; College of Life Science and Technology, Huazhong Agricultural University, Wuhan 430070, China; National Key Laboratory of Agricultural Microbiology, Huazhong Agricultural University, Wuhan 430070, China; College of Life Science and Technology, Huazhong Agricultural University, Wuhan 430070, China; National Key Laboratory of Agricultural Microbiology, Huazhong Agricultural University, Wuhan 430070, China; College of Life Science and Technology, Huazhong Agricultural University, Wuhan 430070, China; National Key Laboratory of Agricultural Microbiology, Huazhong Agricultural University, Wuhan 430070, China; Provincial Key Laboratory of Plant Pathology of Hubei Province, College of Plant Science and Technology, Huazhong Agricultural University, Wuhan 430070, China; National Key Laboratory of Agricultural Microbiology, Huazhong Agricultural University, Wuhan 430070, China; Provincial Key Laboratory of Plant Pathology of Hubei Province, College of Plant Science and Technology, Huazhong Agricultural University, Wuhan 430070, China; National Key Laboratory of Agricultural Microbiology, Huazhong Agricultural University, Wuhan 430070, China; Provincial Key Laboratory of Plant Pathology of Hubei Province, College of Plant Science and Technology, Huazhong Agricultural University, Wuhan 430070, China; National Key Laboratory of Agricultural Microbiology, Huazhong Agricultural University, Wuhan 430070, China; National Key Laboratory of Agricultural Microbiology, Huazhong Agricultural University, Wuhan 430070, China; College of Life Science and Technology, Huazhong Agricultural University, Wuhan 430070, China; National Key Laboratory of Agricultural Microbiology, Huazhong Agricultural University, Wuhan 430070, China; Provincial Key Laboratory of Plant Pathology of Hubei Province, College of Plant Science and Technology, Huazhong Agricultural University, Wuhan 430070, China

## Abstract

Thiolation, a post-transcriptional modification catalyzed by Uba4-Urm1-Ncs2/Ncs6 pathway in three specific transfer RNAs (tRNAs), is conserved from yeast to humans and plays an important role in enhancing codon–anticodon interaction and translation efficiency. Yet, except for affecting effector secretion, its roles in plant pathogenic fungi are not fully understood. Here, we used *Magnaporthe oryzae* as a model system to illustrate the vital role of s^2^U_34_ modification on the appressorium-mediated virulence. The absence of tRNA thiolation leads to diminished translation elongation at AAA/CAA/GAA but not their synonymous codons, resulting in reduced levels of key proteins enriched in these codons, which are critical for appressorium development and function. Importantly, overexpressing these proteins can partially mitigate the defects resulting from *NCS2* deletion. Our study sheds light on the s^2^U_34_ modification’s role in plant pathogenic fungi, enhancing our understanding of translational control beyond effector secretion.

## Graphical Abstract

**Graphical Abstract:**
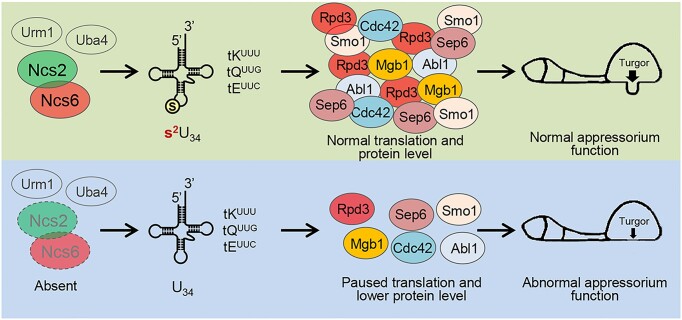

## Introduction

The filamentous fungus *Magnaporthe oryzae* is the causal agent of rice blast, a highly destructive disease affecting cultivated rice worldwide. Through decades of study, *M. oryzae* has become a model fungus for understanding the physiological and pathogenic molecular mechanisms in plant pathogenic fungi (1). The infection process of *M. oryzae* begins when its conidium adheres to the host surface. This is followed by the germination of the conidium, forming a germ tube that eventually differentiates into a dome-shaped cell known as the appressorium. During the development of the appressorium, conidial reserves such as glycogen and lipids are broken down through autophagy, crucial for the formation of the melanized appressorium (2–4). As hydrostatic turgor pressure builds up, the cytoskeleton within the appressorium undergoes reorganization and repolarization, forming a septin ring and then a penetration peg. This structure enables the fungus to breach the host cell (5–8). It is well-established that the cAMP, Pmk1-MAPK and target of rapamycin (TOR) signaling pathways, by regulating the expression of genes, play significant roles in these processes (1,9,10). However, the role of translational regulation during appressorium development is not clear.

Transfer RNA (tRNA) is a key component of the translation machinery, composed of ∼55–95 nucleotides (11). These molecules undergo extensive post-transcriptional modifications, ranging from simple methylations to more complex side chain additions to nucleosides (11). Simple modifications, such as methylations on the ribose or base, typically occur through single enzymatic reactions. These modifications constitute the majority of the modified bases in tRNA and are found nearly ubiquitously across the tRNA molecule, particularly in the D- and T-arms (12). In contrast, complex modifications, which require the sequential action of multiple enzymes, are predominantly located in the anticodon loop (13). These modifications are required for the folding, stability, and functionality of the tRNAs (14).

In the wobble position of tRNA, uridine undergoes complex modifications with various groups. Two distinct sets of modifications occur at U_34_ in the corresponding cytosolic tRNAs for lysine (tK^UUU^), glutamine (tQ^UUG^) and glutamate (tE^UUC^). These modifications include thiolation at the 2-carbon (resulting in s^2^U), and the addition of a methoxycarbonylmethyl side chain at the 5-carbon (forming mcm^5^U). The concurrent presence of these modifications gives rise to mcm^5^s^2^U, one of the most intricate tRNA modifications (15–17). In yeast, the s^2^U_34_ modification is generated through an evolutionarily conserved pathway involving Urm1, Uba4 and Ncs2/Ncs6 complex. Uba4 initially adenylates Urm1 and then converts it into Urm1-thiocarboxylate by transferring a sulfur group from cysteine. The Ncs2/Ncs6 complex subsequently binds to tRNA, adenylates U_34_ at the 2-position, and facilitates the replacement of oxygen with sulfur from Urm1-thiocarboxylate (15,18–21). In *Caenorhabditis elegans* and humans, homologues of Ncs2 and Ncs6 are also essential for this specific modification (22,23).

The s^2^U_34_ modification restricts its base-pairing capability, prevents misreading of near-cognate codons, and enhances the thermostability of codon–anticodon interactions (24–26). A loss of this modification leads to translational defects, including diminished binding to the ribosomal A site, increased ribosomal pausing at corresponding codons, and ribosomal frameshifting (27–32). In budding yeast, tRNA thiolation is crucial for maintaining a proper balance in amino acid utilization for nucleotide synthesis and metabolic homeostasis (33,34). In pathogenic baker’s yeast and human commensal pathogen *Candida albicans*, tRNA thiolation acts as a key mediator of fungal virulence (35). Yeast and worm mutants lacking this modification exhibit temperature sensitivity, endogenous protein aggregation and disruptions in protein homeostasis (22,29). In *Arabidopsis thaliana*, impaired tRNA thiolation leads to a reduction in lateral root density and compromised root hair development (36,37). Recently, it is shown that this modification is also required for the protection of *Arabidopsis* from infection by the pathogenic bacterium *Pseudomonas syringae* (38).

However, the biological significance of tRNA thiolation in plant pathogenic fungi has remained elusive until recently. A recent pivotal study showed that in *M. oryzae*, tRNA thiolation is essential for the translation of unconventionally secreted cytoplasmic effectors, which are enriched in AAA/CAA/GAA codons (39). The loss of Urm1 and Uba4, key sulfur carriers that function upstream of Ncs2 and Ncs6 in the s^2^U_34_ modification pathway, leads to a disruption in the secretion of these effectors into the biotrophic interfacial complex (BIC), thereby hindering biotrophic growth within host cells. This work has been groundbreaking, illuminating the role of tRNA thiolation in the secretion of cytoplasmic effector messenger RNAs (mRNAs) and significantly advancing our field.

In this study, we identified the Ncs2/Ncs6 complex in *M. oryzae*, as a thiolase contributing to cytosolic tRNA thiolation modification, complementing the established role of the Uba4-Urm1 system. We found that deletion of *NCS2* or *NCS6* leads to complete loss of tRNAs thiolation in tK^UUU^, tQ^UUG^, tE^UUC^, and reduced translation elongation rate on cognate AAA/CAA/GAA codons. Building upon the foundational insights provided by Li *et al*. (39), our study sought to delve deeper into the role of tRNA modification and codon usage in the functionality of appressoria, a critical infection structure in *M. oryzae*. Our research expands on the current understanding by demonstrating that the s^2^U_34_ modification is required for the maturation and penetration of appressoria in the pathogenicity of plant pathogenic fungi. By integrating the seminal findings of Li *et al.*with our own, we present a comprehensive view of how tRNA thiolation influences the pathogenicity of *M. oryzae*.

## Materials and methods

### Fungal strains and growth conditions

The *M. oryzae* field isolate strain P131 was used as the wild type. All strains used in this study (Supplementary Table S1) were cultured on Oatmeal Tomato Agar (OTA) plates at 28°C. For RNA and protein extraction, all strains were cultured in liquid complete medium (CM) for 36 h at 28°C. Colony growth observation and conidiation measurement were performed as described previously (40). Conidia from 7-day-old colonies cultured on OTA plates were washed down with 0.025% Tween-20 for inoculation. For rapamycin and diamide sensitivity analysis, strains were inoculated onto the CM plates added with 25 ng/ml rapamycin or 0.5 mM, 1.0 mM, 1.5 mM diamide and cultured at 28°C. The colony diameters were measured at 5 days post inoculation (dpi) to calculate growth reduction rates (40).

### Gene deletion and complementation

For the deletion of *NCS2* and *NCS6*, a gene displacement strategy through split-polymerase chain reaction (PCR) was used as previously described (41). The deletion transformants were selected by 250 μg/ml hygromycin B (Roche) and confirmed by PCR using the *NCS2* or *NCS6* gene-up/gene-down, LCK/HCK-up, RCK/HCK-down primer pairs (Supplementary Figure S1A and B, and Supplementary Table S2). For complementation, the 1.5 kb promoter region and gene-coding region were inserted into pGTN to construct the complementation vectors, pGTN-*NCS2* and pGTN-*NCS6* (Supplementary Table S3), which were transformed into the Δ*ncs2* or Δ*ncs6* mutants, respectively. The complementation transformants were selected by 400 μg/ml neomycin (Amresco) and confirmed by PCR using *NCS2* or *NCS6* gene-up/gene-down primer pairs.

### Virulence test and infection process observation

One-month-old rice seedlings (*Oryza sativa* cv. LTH) and one-week-old barley seedlings (*Hordeum vulgare* cv. E9) were used for virulence test. The rice seedlings were sprayed with conidia suspensions (2 × 10^5^ conidia/ml in 0.025% Tween-20) and then incubated with full humidity at 28°C in dark (before 36 hpi) and light (after 36 hpi). The lesions were photographed and measured at 5 dpi. To test the lesion expansion, we scratched the rice leaves with a punch to make slight wounds on the surfaces of the leaves, and then put mycelium piece on each wound of the rice leaves. The incubated conditions are identical to those described above. The lesions were photographed and measured at 5 dpi. For the observation of the infection process, conidia suspensions (2 × 10^5^ conidia/ml) of different strains were dropped onto the lower barley epidermis, which were then incubated in a dark chamber with full humidity at 28°C. The infection process was observed at 24 and 30 hpi under a microscope (Ni90, Nikon).

### Appressorium cytorrhysis and glycogen/lipid utilization assay

To detect turgor pressure of appressorium, cytorrhysis assay was performed by dropping conidia suspensions (5 × 10^5^ conidia/ml) onto hydrophobic coverslips and incubated them in dark and high humidity at 28°C. After 24 h, varying concentrations [25%, 30% and 35% (*w/v*)] polyethylene glycol (PEG) 8000 solutions were added on the appressoria that formed on the coverslips and treated for 5 min. Percentages of the collapsed appressoria were calculated and photographed under a microscope (Ni90, Nikon). At least 100 conidia were examined for each concentration.

To observe glycogen and lipid droplet utilization, conidia suspensions dropped onto hydrophobic coverslips with incubate conditions identical to those described above, were then stained with staining solution for 10 min at 0, 2, 4, 8, 12 and 18 hpi. KI/ I_2_ solution (60 mg/ml KI, 10 mg/ml I_2_) was used for glycogen staining, and Nile Red (2.5 mg/ml, Sigma–Aldrich) was used for lipid droplet staining. The stained germinating conidia and appressoria were calculated and photographed under a microscope (Ni90, Nikon).

### Autophagy process observation

A GFP-Atg8 fusion expressing vector was transformed into the wild-type, Δ*ncs2* and Δ*ncs6* strains. The hyphae of the above transformants were cultured in liquid CM at 28°C for 48 h, followed by transferring to nitrogen deficient liquid minimum medium (MM-N) and induced for another 5 h, then were observed through the fluorescence signal of GFP-Atg8 under a confocal microscope (Leica Microsystems). To observe the autophagosomes number, conidia suspensions (1 × 10^5^ conidia/ml) were dropped onto hydrophobic plastic cover glasses and incubated for 2 h or 4 h. The numbers of autophagosomes were observed and counted under a confocal microscope.

### Septin ring observation

A Sep6-GFP fusion expressing vector was transformed into the wild-type, Δ*ncs2* and Δ*ncs6* strains. The conidia suspensions of the transformants were dropped onto hydrophobic coverslips and incubated for 24 h, and then the appressoria were observed under confocal microscope. The fluorescence intensity was measured by Image J software.

### Western blotting

For protein extraction, the mycelia cultured in liquid CM for 36 h were collected, ground into powder in liquid nitrogen and resuspended in protein extraction buffer (Biyuntian) added with 1 mM Phenylmethylsulfonyl fluoride (PMSF) (Sigma–Aldrich). For Co-immunoprecipitation (CoIP) assay, total proteins were incubated for 2 h at room temperature after addition of anti-Green fluorescent protein (anti-GFP) beads (Bimake), and then eluted from the beads by adding elution buffer [0.1% RapiGest in 50 mM tetramethylammonium bromide (TMAB)]. In the following western blot, anti-FLAG antibody (1:5000; Yeasen) or anti-GFP antibody (1:5000, Yeasen) was used as the primary antibody, and anti-mouse horseradish peroxidase (1:10 000, Abmart) was used as the secondary antibody. For autophagy analysis, mycelia of strains under nitrogen deficient treatment described above for 0, 2 or 5 h was collected to extract proteins. Western blot was performed with anti-GFP antibody (1:5000, Yeasen) or anti-glyceraldehyde phosphate dehydrogenase (anti-GAPDH) antibody (1:5000, Dia-an), and anti-mouse horseradish peroxidase (1:10 000, Abmart). For Rpd3 protein level detection, anti-Rpd3 antibody (kindly provided by Dr Qun He, China Agricultural University) was used as the primary antibody; for Abl1 protein level detection, Abl1-hemagglutinin (Abl1-HA) fusion protein was detected with anti-HA antibody (1:5000, Abclonal); for Cdc42/Smo1/Mgb1/Sep6 protein level detection, Cdc42/Smo1/Mgb1/Sep6 proteins fusion with GFP were detected using anti-GFP antibody. For all the above six proteins, the anti-mouse horseradish peroxidase (1:10 000, Abmart) was used as the secondary antibody.

### RNA extraction, quantitative reverse transcriptase-polymerase chain reaction and northern blot

RNA extraction and quantitative reverse transcriptase-polymerase chain reaction (qRT-PCR) were performed based on previously described protocols, with slight modifications (42). For RNA extraction, mycelia cultured in liquid CM for 36 h were collected using vacuum filtration. The frozen tissues were subsequently ground into fine powders using a mortar and pestle in the presence of liquid nitrogen. Total RNA was then extracted using a Direct-zol™ RNA MiniPrep Plus kit (Zymo Research), according to the manufacturer’s instructions. The obtained RNA was quantified using a Nanodrop spectrophotometer. For qRT-PCR assay, complementary DNA (cDNA) was obtained by reverse transcription using a M5 HiPer First Strand DNA Synthesis kit (Mei5 Biotechnology), following the instructions. The resulting cDNA was then subjected to real-time PCR analysis. For Northern blotting of tRNAs, 10 μg total RNA samples with 2 × TBE loading buffer were loaded onto a 10% polyacrylamide gel with 7 M urea, 0.5 × TBE and 2 μg/ml (*N*-acryloylamino)phenylmercuric chloride (APM) to separate the thiolated and un-thiolated RNAs by their molecular weight at 200 V in 0.5 × Tris-Borate-ethylenediaminetetraacetic acid (TBE) buffer (45 mM Tris, 45 mM borate, 1 mM ethylenediaminetetraacetic acid, pH 8.0). Then the separated RNAs were transferred onto Hybond™-N + positive charged nylon membranes (GE Healthcare) using a semi-dry transformer (Bio-Rad) in 0.5 × TBE buffer at a current of 300 mA for 30 min. After ultraviolet (UV) crosslinking at 120 000 μJ/cm^2^ in a Stratalinker UV crosslinker (UVP Laboratory Products), the membrane was probed with biotin-labeled probes against specific tRNAs, which was subsequently developed using the Chemiluminescent Nucleic Acid Detection Module kit (Thermo Fisher Scientific).

### Polysome profiling

Mycelia cultured in liquid CM for 36 h and appressoria on hydrophobic surface for 6 h were collected. Cell lysate was prepared from the grounded tissue using a polysome extraction buffer [200 mM Tris-HCl (pH7.5), 200 mM KCl, 35 mM MgCl_2_, 100 μg/ml cycloheximide (CHX), 15 mM Beta-Mercaptoethanol (BME), 1 mM dithiothreitol (DTT), 20 U/ml RNase inhibitor (Thermo Fisher)]. RNA concentration was measured using a Nanodrop spectrometer, and 10 optical density (OD) of cell lysate in a 200 μl volume was used for each experiment, with 10% reserved as the ‘Input’ sample; 15% and 50% (*w/v*) sucrose solutions were prepared using a polysome buffer (40 mM Tris-HCl, pH7.5, 20 mM KCl, 10 mM MgCl_2_). Linear 15–50% sucrose gradients were generated using a BioComp Gradient Master (Biocomp Instruments). Then the lysate was loaded onto the sucrose gradient and centrifuged at 38 000 rpm at 4°C for 3 h in a swinging bucket rotor (Beckman SW41). Post-centrifugation, the gradient was fractionated and collected using a Gradient Station (BioComp), equipped with a UV monitor to detect absorbance at 254 nm.

To assess the translation of specific mRNA, RNAs were extracted from both the ‘Input’ samples and gradient fractions. An equal volume of TRIzol reagent and chloroform were added to each sample, followed by shaking on a 3D shaker at 25°C for 5 min and centrifugation at 12 000 g at 4°C for 15 min. The supernatant was transferred to a new tube and precipitated with an equal volume of isopropanol, supplemented with 1 μl GlycoBlue (Thermo Fisher). The RNA precipitate was washed twice with 75% ethanol and resuspended in 8 μl RNase-free water for subsequent qRT-PCR analysis. Values from each fraction were normalized to the ‘Input’ sample and represented as a percentage of the input (Input%).

### Ribosome profiling and data analysis

The Ribosome profiling (Ribo-seq) was conducted according to previously published protocols, with some modifications (43,44). The libraries were generated from the wild-type (WT) and Δ*ncs2* strains with culture conditions identical to those described in the section of polysome profiling. Cell lysates were prepared with a polysome extraction buffer [200 mM Tris-HCl (pH7.5), 200 mM KCl, 35 mM MgCl_2_, 100 μg/ml CHX, 15 mM BME, 1 mM DTT]. A 100 μl aliquot of the cell lysate was reserved as the ‘Input’ sample for RNA extraction. Meanwhile, 200 μl of the cell lysate were digested with RNase I (Life Technologies) for 1 h at 25°C, following by adding 1 μl SUPERase*In RNase Inhibitor (Life Technologies) to terminate the reaction. Monosomes were collected through sucrose gradients fractionation. RNA was then extracted using TRIzol reagent (Life Technologies) and separated on a 16% denaturing gel. Ribosome-protected fragments (RPFs), ∼26–34 nucleotides in length, were isolated from the gel. The 3′ ends of these RPFs were repaired with T4 PNK (NEB). Adaptors were then ligated to the RPFs using T4 RNA Ligase 2, truncated KQ (NEB), and the reaction was incubated at 22°C for 3 h. The ligated RPFs were purified and reverse transcribed using SuperScript III Reverse Transcriptase (Thermo Fisher). The resulting cDNAs were further purified and circularized using CircLigase ssDNA Ligase (Lucigen) for 2 h at 60°C. The cleaned cDNAs were amplified using Q5 High-Fidelity 2X Master Mix (NEB) and purified by using AMPure XP Beads (Beckman Coulter). Finally, the libraries were sequenced on the Illumina Novaseq 6000 platform.

For the analysis of Ribo-seq data, raw sequencing reads were first trimmed of adaptors and random sequences using Cutadapt (http://dx.doi.org/10.14806/ej.17.1.200) (45), following by quality control to remove low-quality reads. Next, Bowtie (46) was employed to eliminate contaminating ribosomal RNA and tRNA sequences before mapping the cleaned reads to the *M. oryzae* genome assembly MG8 using STAR v2.7 (47). Only reads uniquely mapped to the genome were retained for further analysis. Specialized metrics like RPF length distribution, 3-nucleotide periodicity and offset detection were assessed through a customized Python pipeline, available upon request from Z.Z. All downstream analyzes used reads with 27–35 nt from frame 0. The RPF abundance for each gene was calculated by above pipeline. DESeq2 package was used to analyze differential gene expression, while ‘clusterProfiler’ R package was employed for Gene Ontology (GO) and Kyoto Encyclopedia of Genes and Genomes (KEGG) analyses. Furthermore, genes with >50 RPFs would be selected to calculate ribosome occupancy ribosome occupancy (29) at A-site to assess the codon decoding rate.

### RNA-seq data analysis

For the analysis of RNA-seq data, raw sequencing reads were first trimmed of illumina adaptors using Trimmomatic (48), following by quality control to remove low-quality reads. The clean reads were then mapped to the *M. oryzae* genome assembly MG8 using STAR v2.7 (47). Only reads uniquely mapped to the genome were retained for further analysis. FeatureCounts (49) was utilized for mRNAs abundance quantification. DESeq2 package was used to analyze differential gene expression, while ‘clusterProfiler’ R package was employed for GO and KEGG analyses.

### Mass spectrometry and proteomic analysis

Mycelia of the WT and Δ*ncs2* strains cultured under identical conditions as specified in the polysome profiling section were harvested using vacuum filtration and immediately snap-frozen in liquid nitrogen. Liquid chromatography-tandem mass spectrometry was performed following the Tandem Mass Tag labeling approach, with bioinformatics analysis conducted by PTM Biolabs in Hangzhou, China. A threshold of 1.2-foldchange and *P*< 0.05 was used to define the differentially expressed proteins. For each differentially expressed group, we calculated the codon frequency (the frequency per 1000 codons) of AAA/CAA/GAA per gene. Functional analyses for the proteomic data followed the same methods used in the Ribo-seq analysis.

### Codon frequency calculation

Codon frequency (numbers per 1000 codons) of AAA/CAA/GAA/AAG/CAG/GAG per gene was calculated by customized Python script.

### Quantification and statistical analysis

Two-tails *t*-tests or Wilcoxon’s signed-rank tests were employed for group comparisons. Correlation analyses were conducted using either Pearson or Spearman tests. A *P*-value of <0.05 was considered statistically significant. Data visualization was accomplished using custom R scripts.

## Results

### The Ncs2/Ncs6 complex is required for tRNA thiolation in *M. oryzae*

**Figure 1.**
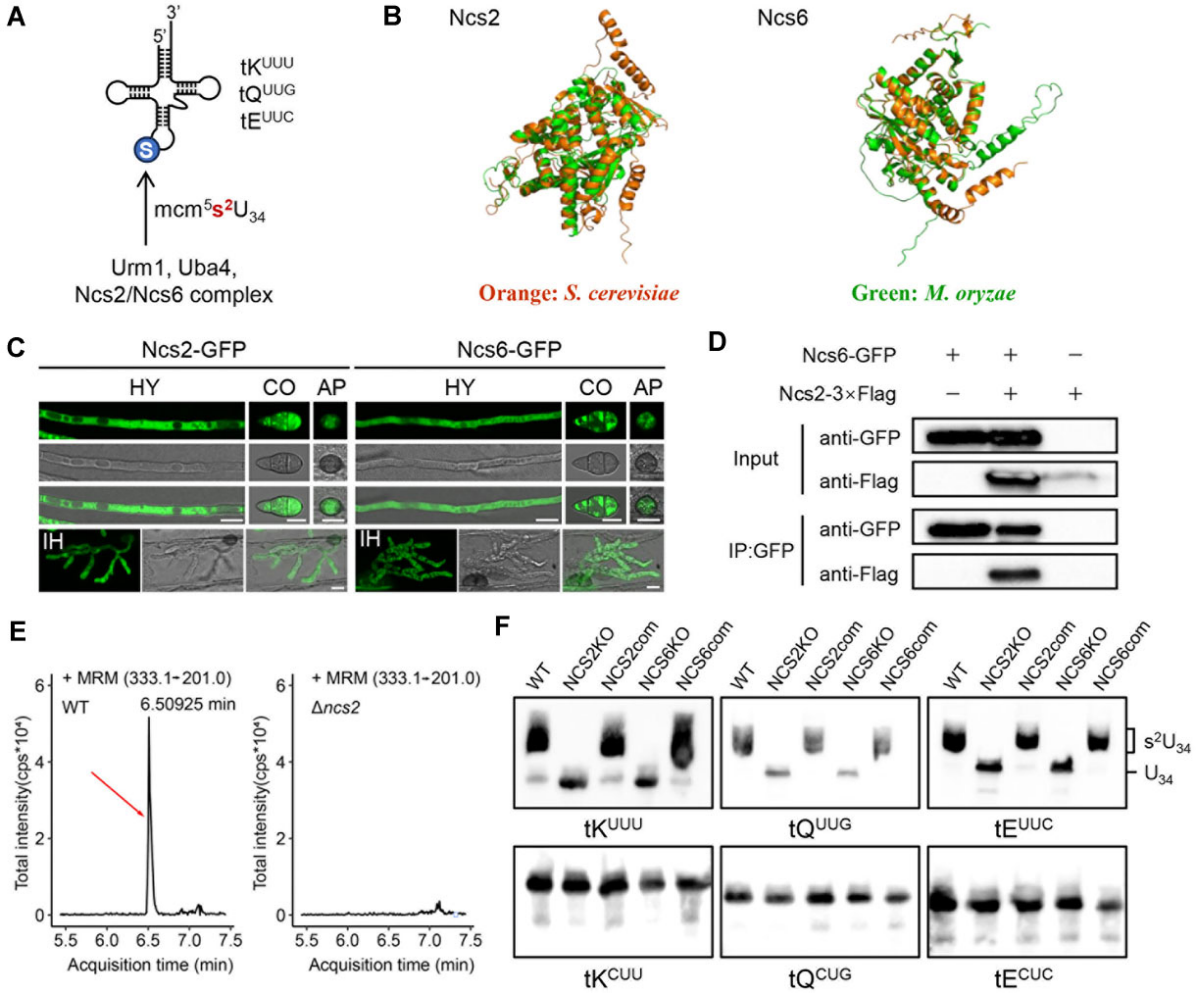
Ncs2/Ncs6 complex is required for tRNA thiolation of *M. oryzae*. (**A**) 2-thiolation of tRNA anticodon wobble U_34_ is mediated by the conserved Ncs2/Ncs6 complex. (**B**) Overlapping analysis of the protein structure of Ncs2 and Ncs6 in *S. cerevisiae* and *M. oryzae*. (**C**) Subcellular localization of Ncs2 and Ncs6 in *M. oryzae*. The hyphae, conidia, appressoria and infection hyphae of transformants expressing Ncs2-GFP or Ncs6-GFP were observed under a confocal microscope. HY, vegetative hyphae; CO, conidium; AP, appressorium; IH, invasive hyphae. Bar = 10 μm. (**D**) CoIP analyses between Ncs2 and Ncs6. Ncs2-3 × Flag and Ncs6-GFP constructs were respectively or co-expressed in the wild-type strain. Total protein was eluted from anti-GFP beads and then detected by anti-Flag or anti-GFP antibody. (**E**) Mass chromatograms of the mcm^5^s^2^U tRNA modification in vegetative mycelia of the wild-type and Δ*ncs2* mutant strain. The y-axes are ion counts; the x-axes are acquisition times. The mcm^5^s^2^U modification peak position is indicated by a red arrow. (**F**) Northern blot analysis of cytoplasmic tRNAs from the wild-type, Δ*ncs2*, Δ*ncs6* and complementary strains. APM gel was used for electrophoresis, in which the thiolation-modified tRNAs migrates much slower than those without thiolation modification. Specific probes against tK^UUU^, tQ^UUG^ and tE^UUC^ were used for northern blot analysis. Specific probes against their cognate tRNAs, tK^CUU^, tQ^CUG^, tE^CUC^ were used for control. WT, wild type; NCS2KO, the *ncs2* knock-out mutant; NCS2com, the *NCS2* complement strain; NCS6KO, the *ncs6* knock-out mutant; NCS6com, the *NCS6* complement strain.

The tRNA thiolation modification is catalyzed by the conserved Ncs2/Ncs6 complex across various species, from yeast to humans (Figure 1A) (22,23,50,51). In *M. oryzae*, the genes MGG_06404 and MGG_04613 encode the orthologues of Ncs2 and Ncs6, respectively. Multiple sequence alignments revealed that Ncs6 is more conserved than Ncs2 across species. Specifically, *M. oryzae* Ncs2 shares 18%–27% identity with its homologs, while Ncs6 protein exhibits 49%–71% amino acid identity (Supplementary Figure S2). However, the structures of both *M. oryzae* Ncs2 and Ncs6 closely resemble those in *Saccharomyces cerevisiae* (Figure 1B). Furthermore, both proteins were primarily localized in cytoplasm, similar to their yeast counterparts (Figure 1C). CoIP experiments confirmed that *M. oryzae* Ncs2 interacts with Ncs6, suggesting they form a complex as in yeast (Figure 1D). These findings strongly suggest that *M. oryzae* Ncs2/Ncs6 complex likely performs similar molecular functions as its yeast counterparts.

To test the effect of the Ncs2/Ncs6 complex on tRNA thiolation in *M. oryzae*, we generated *NCS2* and *NCS6* deletion mutants using a gene displacement strategy (41), verified by PCR (Supplementary Figure S1A and B). We performed HPLC-coupled mass spectrometry on total tRNA isolated from wild-type and Δ*ncs2* mutant, and found that deletion of *NCS2* leads to complete loss of thiolation modification in total tRNA (Figure 1E). To further test thiolation modification status in individual tRNA, we performed northern blotting using denaturing PAGE gel supplemented with APM (Figure 1F). As seen in other organisms, tK^UUU^, tQ^UUG^, tE^UUC^ showed reduced mobility in the wild-type strain due to the affinity of the mercuric compound for the thiolated tRNAs. Interestingly, a small portion of tK^UUU^ was non-thiolated, whereas all tQ^UUG^, tE^UUC^ were thiolated. Importantly, all tK^UUU^, tQ^UUG^, tE^UUC^ migrated faster in *NCS2* and *NCS6* deletion mutants, which could be reversed by reintroducing wild-type *NCS2* or *NCS6* into the corresponding mutants. In contrast, deleting Ncs2 or Ncs6 did not affect the mobility of tK^CUU^, tQ^CUG^, tE^CUC^. These results unequivocally demonstrate that the Ncs2/Ncs6 complex is essential for tRNA thiolation in *M. oryzae*, consistent with findings in other eukaryotes (Figure 1F).

Interestingly, *NCS2* and *NCS6* exhibited a dynamic expression pattern during appressorium development. Their levels increased, peaked at around 3 or 6 h post inoculation (hpi), decreased, and then rose again at 44_hpi (Supplementary Figure S3). This result suggests that *NCS2* and *NCS6* may play a crucial role in appressorium maturation by mediating tRNA thiolation in *M. oryzae*.

### tRNA thiolation affects hyphae growth, conidia production and stress response of *M. oryzae*

**Figure 2.**
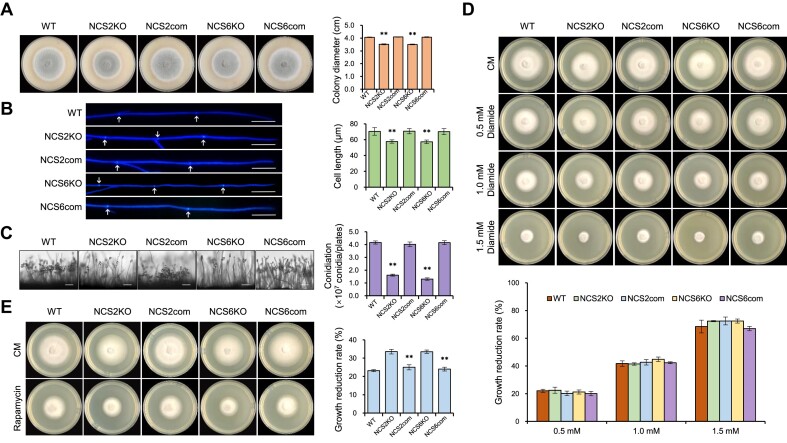
Ncs2/Ncs6-mediated tRNA thiolation is required for growth, conidiation, and rapamycin stress response. (**A**) The colony morphology and the colony diameters of different strains grown on OTA plates at 28°C for 5 days. The data represent mean values with standard deviations of three biological replicates. Asterisks denote significant differences compared to WT (*P*< 0.01, Student’s *t*-test, two-tailed). (**B**) Hypha tips of different strains were stained by calcofluor white (CFW), and then the length of hypha apical cells was measured. Cell septa are indicated with arrows. Bar = 20 μm. The data represent mean values with standard deviations of 50 hypha apical cells. Asterisks denote significant differences compared to WT (*P*< 0.01, Student’s *t*-test, two-tailed). (**C**) Conidiophore of different strains grown on OTA plates were observed under a light microscopy. Bar = 50 μm. And Conidiation of different strains were calculated. Conidia on per OTA plates were washed with 30 ml of water and counted using a hemocytometer. The data represent mean values with standard deviations of three biological replicates. Asterisks denote significant differences compared to WT (*P*< 0.01, Student’s *t*-test, two-tailed). WT, wild type; NCS2KO, the *ncs2* knock-out mutant; NCS2com, the *NCS2* complement strain; NCS6KO, the *ncs6* knock-out mutant; NCS6com, the *NCS6* complement strain. (**D**) Assay for diamide sensitivity. Strains were cultured on CM plates containing 0.5, 1.0 and 1.5 mM diamide at 28°C for 5 days. The growth reduction rates were calculated by colony diameter. The data represent mean values with standard deviations of three biological replicates. (**E**) Assay for rapamycin sensitivity. Strains were cultured on CM plates containing 25 ng/ml rapamycin at 28°C for 5 days. The growth reduction rates were calculated by colony diameter. The data represent mean values with standard deviations of three biological replicates. Asterisks denote significant differences compared to WT (*P*< 0.01, Student’s *t*-test, two-tailed).

To determine whether Ncs2/Ncs6-mediated tRNA thiolation is important for *M. oryzae*, we first compared hyphal growth between wild-type and tRNA thiolation-deficient strains. Deleting *NCS2* and *NCS6* led to reduced hyphal growth (Figure 2A), which was attributed to shortened cell lengths of apical hyphae (Figure 2B). Moreover, conidia production in the Δ*ncs2* and Δ*ncs6* mutants was approximately one-third of that in wild-type strains (Figure 2C). These defects were rescued by ectopic expression of *NCS2* and *NCS6* in the corresponding mutants, indicating that tRNA thiolation is critical for vegetative growth and asexual spore development in *M. oryzae*.

Previous studies have shown that modifications on U_34_ are associated with stress response (18,35,52,53). However, compared to the wild-type, the *NCS* mutants exhibited similar sensitivity to oxidizing reagents such as diamide and H_2_O_2_ (Figure 2D and Supplementary Figure S4A), and to cell wall integrity-disturbing agents like CFW and Congo red (Supplementary Figure S4B). In contrast, both mutants were more sensitive to rapamycin (Figure 2E), a TOR pathway inhibitor, similar to observations in other species (18,35). These results suggest that tRNA thiolation is required for stress response in *M. oryzae*.

### tRNA thiolation influences *M. oryzae* virulence by affecting appressorium-meditated cuticle penetration

**Figure 3.**
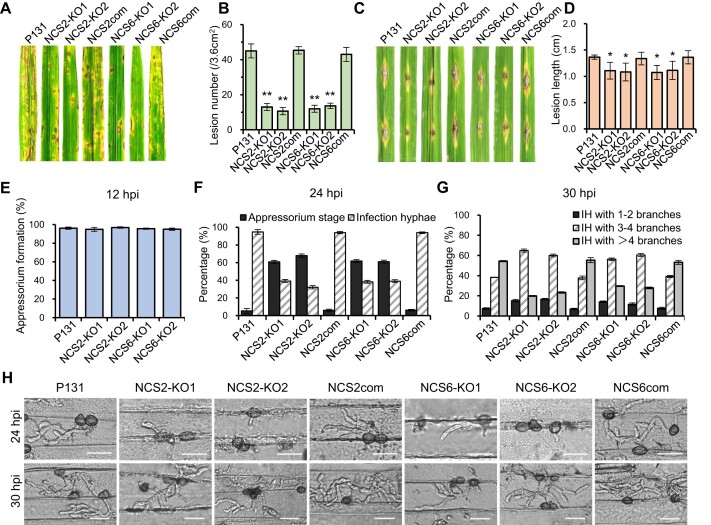
Ncs2/Ncs6 complex mediated tRNA thiolation is required for full virulence of *M. oryzae*. (**A**) Conidia suspension (2 × 10^5^ conidia/ml) of the wild-type, Δ*ncs2*, Δ*ncs6* and complement strains were sprayed onto rice leaves and incubated for 5 days. (**B**) The lesion number counting and statistics. The data represent mean values with standard deviations of three biological replicates. Asterisks denote significant differences compared to WT (*P*< 0.01, Student’s *t*-test, two-tailed). (**C**) Conidia suspension (2 × 10^5^ conidia/ml) of the wild-type, Δ*ncs2*, Δ*ncs6* and complement strains were dropped onto the wounds of scratched rice leaves and incubated for 5 days. (**D**) The lesion length measuring and statistics. The data represent mean values with standard deviations of three biological replicates. Asterisks denote significant differences compared to WT (*P*< 0.01, Student’s *t*-test, two-tailed). (**E**) Statistic of the appressoria formation rate on hydrophobic plastic cover glasses. (**F**) Statistic on the percentages of appressoria with and without invasive hyphae after strains inoculated to barley epidermal cells 24 h. (**G**) Statistic on the percentages of invasive hyphae at different stages after strains inoculated to barley epidermal cells 30 h. IH, invasive hypha. The data represent mean values with standard deviations of three biological replicates, and at least 50 conidia or appressoria were counted for each time. (**H**) The invasive hyphae of the wild-type, Δ*ncs2*, Δ*ncs6*, and complement strains in barley epidermal cells at 24 and 30 hpi. Bar = 20 μm. P131, the wild-type; NCS2KO, the *ncs2* knock-out mutant; NCS2com, the *NCS2* complement strain; NCS6KO, the *ncs6* knock-out mutant; NCS6com, the *NCS6* complement strain.

Does tRNA thiolation influence the virulence of *M. oryzae*? We found that the lesion number caused by the Δ*ncs2* and Δ*ncs6* mutants on rice leaves were ∼30% of those caused by the wild-type strain (Figure 3A and B), which were restored by expressing *NCS2* or *NCS6* in the corresponding mutants. Consistent with previous study, the loss of tRNA thiolation due to deleting *NCS* genes led to decreased disease lesions on wounded rice leaves (Figure 3C and D). Together, these findings indicate that tRNA thiolation is required for full virulence in *M. oryzae*.

To understand how tRNA thiolation affects *M. oryzae* virulence, we examined the major infection steps of the infection process. By 12 hpi, appressorium formation rate was comparable between the wild-type and *NCS* mutants, suggesting that tRNA thiolation has little or no effect on appressorium development (Figure 3E). However, the appressorium-mediated cuticle penetration was delayed in Δ*ncs2* and Δ*ncs6* mutants (Figure 3F–H). For instance, at 24 hpi, over 90% conidia from the wild-type strain had penetrated host cells and formed invasive hyphae, in contrast to only about 40% in the Δ*ncs2* and Δ*ncs6* mutants. Additionally, the Δ*ncs2* and Δ*ncs6* mutants did not trigger reactive oxygen species accumulation in host cells (Supplementary Figure S4C), nor did they show increased sensitivity to oxidative stresses (Figure 2D and Supplementary Figure S4B). These results indicate that appressorium-mediated cuticle penetration is significantly impaired in tRNA thiolation-deficient strains.

### tRNA thiolation plays a pivotal role in functionality of appressorium

**Figure 4.**
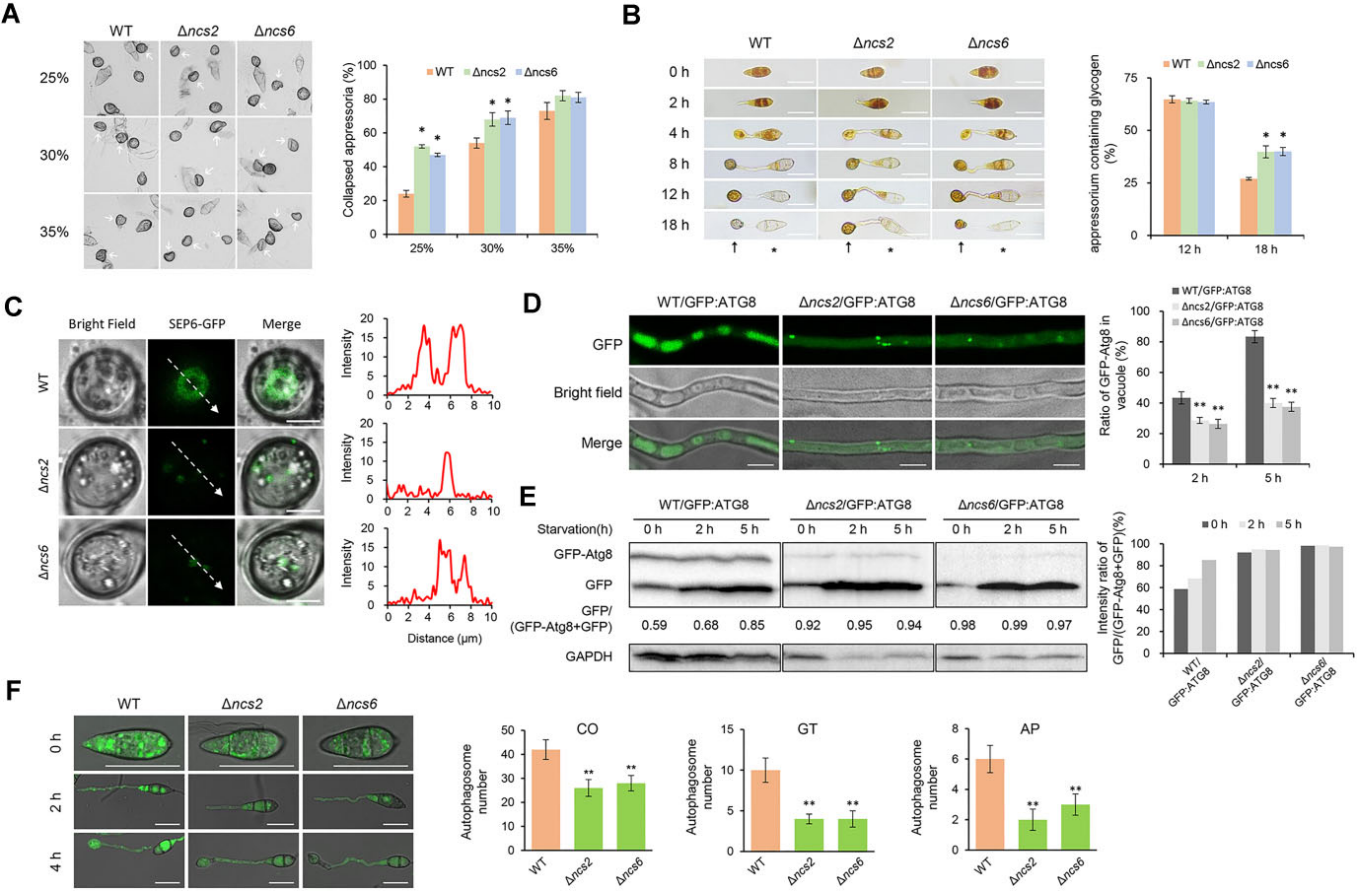
Ncs2/Ncs6 complex-mediated tRNA thiolation is required for appressorium functionality of *M. oryzae*. (**A**) Cytorrhysis assay for appressorium turgor pressure detection. Conidia suspensions (5 × 10^5^ conidia/ml) were dropped on hydrophobic cover glass and cultured for 24 h, and then treated with different concentrations of PEG8000 solutions for 5 min. Arrows indicate collapsed appressoria. Percentages of the collapsed appressoria were calculated. The data represent mean values with standard deviations of three biological replicates, and at least 50 conidia were counted for each time Asterisks denote significant differences compared to WT (*P*< 0.05, Student’s *t*-test, two-tailed). (**B**) Staining assay to detect the utilization of glycogen during appressorium formation. Conidia suspensions dropped on hydrophobic cover glass were stained with I_2_ /KI solution at different time points. Stained glycogen exhibited yellowish-brown color. * indicates conidia, ↑ indicates appressoria. Bar = 20 μm. Percentages of the stained appressoria were calculated. The data represent mean values with standard deviations of three biological replicates, and at least 50 conidia were counted for each time. Asterisks denote significant differences compared to WT (*P*< 0.05, Student’s *t*-test, two-tailed). (**C**) Septin ring observation. Appressoria of strains that expressing Sep6-GFP were observed under a confocal microscope. Line scan graph was generated at the indicated positions (arrow) to confirm the localization of Sep6. Bar = 5 μm. (**D**) Autophagic processes observation under nitrogen starvation. Hyphae of the wild-type, Δ*ncs2* and Δ*ncs6* expressing GFP-Atg8 were cultured under nitrogen starvation for 5 h. The subcellular localizations of autophagosomes were observed under a confocal microscope, and the percentages of cells that GFP-Atg8 transferred into vacuoles were calculated. Bar = 5 μm. The data represent mean values with standard deviations of three biological replicates, and at least 30 hyphae were counted for each time. Asterisks denote significant differences compared to WT (*P*< 0.01, Student’s *t*-test, two-tailed). (**E**) Western blot analysis for autophagy intensity assessment. The amounts of free GFP proteins and intact GFP-Atg8 proteins were detected by anti-GFP antibody after the strains were cultured under nitrogen starvation for 0, 2 or 5 h. Anti-GAPDH antibody was used for the control. The intensity of autophagy was estimated by calculating the signal intensity ratio of GFP/(GFP + GFP:Atg8). (**F**) The number of autophagosomes during appressorium formation. Conidia of strains that expressing GFP-Atg8 germinated (2 h) and formed appressorium (4 h) on hydrophobic cover glass, and then were observed under a confocal microscope. Bar = 20 μm. The numbers of autophagosomes were counted. Data represent mean values with standard deviations of three biological replicates. Asterisks denote significant differences compared to WT (*P*< 0.01, Student’s *t*-test, two-tailed).

Typically, penetration defects are caused by insufficient turgor pressure within the appressorium. Indeed, appressoria turgor pressure in the Δ*ncs2* and Δ*ncs6* mutants was significantly lower than that in the wild-type strain. For instance, in a 25% PEG8000 solution, the number of collapsed spores in the Δ*ncs2* and Δ*ncs6* mutants were more than twice that of the wild-type strain (Figure 4A). Consist with the reduced turgor pressure, glycogen utilization was significantly delayed in the Δ*ncs2* and Δ*ncs6* mutants during appressorium development (Figure 4B). At 18 hpi, only 25% glycogen remained in wild-type cells, compared to about 45% in *NCS* mutants. Due to impaired turgor pressure accumulation, the formation of the septin ring was significantly impaired in the thiolation-defective mutants (Figure 4C). In the wild-type strain, SEP6-GFPs formed a ring-shaped structure, while they formed several dispersed granules within the appressorium in the Δ*ncs2* and Δ*ncs6* mutants. These data indicate tRNA thiolation is critical for turgor pressure generation and penetration peg function.

Given that autophagy regulates glycogen utilization during appressorium formation (54), and that the Δ*ncs2* and Δ*ncs6* mutants are more sensitive to rapamycin (55,56), we hypothesized that tRNA thiolation regulates appressorium functionality by affecting autophagy. To test this hypothesis, we introduced GFP-tagged ATG8 in *M. oryzae* to label autophagosomes. After 5 h of nitrogen starvation, ATG8-labeled autophagosomes had been successfully delivered into vacuoles in over 80% of wild-type hypha cells, while this number decreased to ∼40% in strains lacking tRNA (Figure 4D). Next, we compared autophagy activity by examining free GFP cleaved from GFP-ATG8 fusion proteins. During nitrogen starvation, relative free GFP levels increased from 0.59 to 0.85 in the wild-type strain, indicating increased autophagy (Figure 4E). In contrast, in the Δ*ncs2* and Δ*ncs6* mutants, free GFP levels maintained high throughout this process (> 0.90), indicating defects in autophagy. Consistent this, the numbers of autophagosomes in the Δ*ncs2* and Δ*ncs6* mutants was significantly lower than in the wild-type strain during appressorium development (Figure 4F). These results indicate that tRNA thiolation is essential for normal autophagy in during *M. oryzae*.

### tRNA thiolation impacts translation in codon-usage dependent manner in *M. oryzae*

Previous studies have shown that tRNA thiolation impacts translation elongation speed on AAA/CAA/GAA codons in nematode, yeast and *C. albicans* (29,35). To determine if this mechanism operates similarly in *M. oryzae*, we performed Ribo-seq experiments. Our assessment of data repeatability, the length distribution of RPFs, origins of RPFs and 3-nucleotide periodicity confirmed the quality of our Ribo-seq data (Supplementary Figure S5A–D), providing a reliable foundation for our studies on translation in *M. oryzae*.

**Figure 5.**
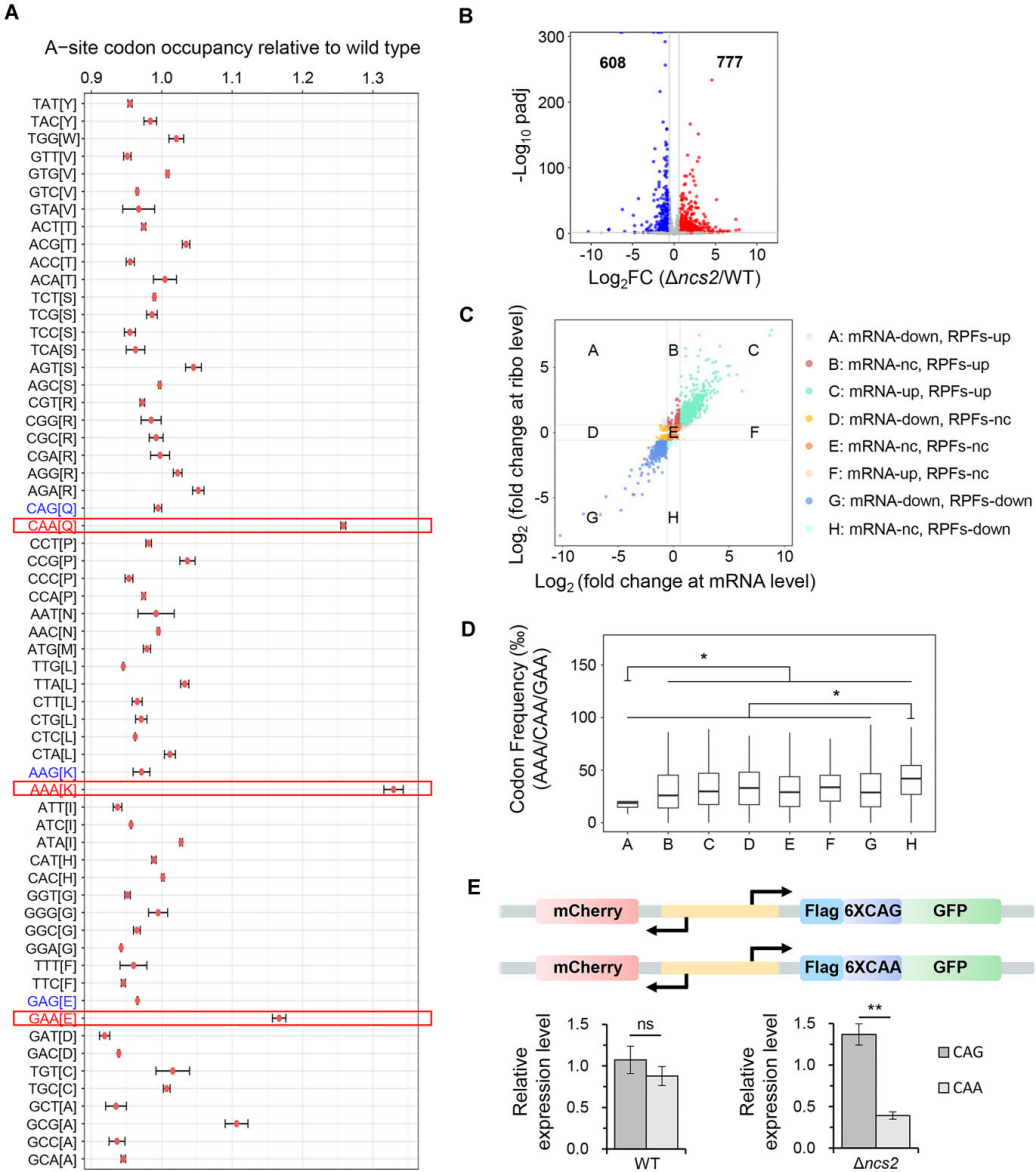
The absence of tRNA thiolation resulted in increased ribosome pausing on AAA/CAA/GAA codons in *M. oryzae*. (**A**) Codon-specific changes at A-site ribosome occupancy in Δ*ncs2* mutant compared with the wild-type. The codons CAA, AAA and GAA are decoded by tRNAs with thiolation, and the codons CAG, AAG and GAG are their synonymous codons. (**B**) Volcano plot of differentially detected RPFs in Δ*ncs2* compared to the wild-type. Right points, log_2_ foldchange ≥ 0.585; left points, log_2_ foldchange≤-0.585; middle points, not significant. *P*< 0.05 (two-tailed *t*-test). n = 2 samples per group. (**C**) Classification of genes based on fold changes of RPFs and mRNAs. The genes with *P*< 0.05 in RNA-seq and Ribo-seq were selected for classification. A: Downregulated in RNA-seq but upregulated in Ribo-seq; B: Non-change in RNA-seq but upregulated in Ribo-seq; C: Upregulated in RNA-seq and Ribo-seq; D: Downregulated in RNA-seq but non-change in Ribo-seq; E: Non-change in RNA-seq and Ribo-seq; F: Upregulated in RNA-seq but non-change in Ribo-seq; G: Downregulated in RNA-seq and Ribo-seq; H: Non-change in RNA-seq but downregulated in Ribo-seq. (**D**) Comparison of AAA/CAA/GAA codons frequency between genes through classification based on fold changes of RPFs and mRNAs. Statistics calculated by Student’s *t*-test. *P < 0.05. (**E**) Sketch of the reporter vectors and the western blot analysis for the fusion protein translation efficiency. The amounts of GFP proteins were detected by anti-GFP antibody. Anti-mCherry antibody was used as control. The expression levels were estimated by calculating the signal intensity of bands. Statistics calculated by Student’s *t*-test. **P< 0.01.

Using Ribo-seq data, we found that deleting *NCS2* resulted in increased ribosome occupancy at CAA/AAA/GAA codons, consistent with observations in other species (Figure 5A). In contrast, ribosome occupancies were comparable between wild-type and Δ*ncs2* strains on synonymous CAG/AAG/GAG codons, which are read by tRNAs without thiolation (Figure 5A). At the gene level, we found that in the Δ*ncs2* mutant, 608 genes showed decreased translation compared to the wild-type strain, while 777 genes exhibited increased translation (Figure 5B and Supplementary Table S4). Both GO analysis and KEGG pathway enrichment analysis revealed that amino acids biosynthesis pathways were significantly enriched among the up-translated genes, suggesting that translation defects caused by deleting *NCS2* may lead to increased amino acid synthesis as a compensatory response (Supplementary Figure S6A and B). These results confirm that tRNA thiolation is important for maintaining global translation profile by directly affecting the translation of specific codons and indirectly by influencing amino acid biosynthesis.

To understand how tRNA thiolation influences gene expression, we classified all significantly affected genes into eight classes (Figure 5C) based on fold changes in RPF and mRNA levels. Genes in class A showed increased RPF levels but decreased mRNA levels, indicating elevated translation efficiency in the Δ*ncs2* mutant. In contrast, genes in class H exhibited decreased RPF levels and unchanged mRNA levels, indicating reduced translation efficiency after deleting *NCS2*. Surprisingly, we found that class A genes have the lowest frequency of AAA/CAA/GAA codons, while class H genes have the highest frequency of these codons (Figure 5D). However, no such trend was observed for their synonymous codons (Supplementary Figure S7A). These findings suggest that tRNA thiolation promotes the translation of genes enriched in AAA/CAA/GAA codons.

To further substantiate our findings, we employed a reporter assay. Both CAA and CAG codons encode glutamine, yet only CAA is decoded by thiolated tRNA. The loss of thiolation led to increased ribosome pausing at CAA codons, but not at CAG codons (Figure 5A). In this assay, we engineered a construct with six consecutive CAA or CAG codons inserted between an N-terminal Flag tag and a C-terminal GFP. The expression of GFP was normalized against mCherry, which was driven by the same bidirectional promoter. Notably, the use of CAA codons led to a significantly reduced GFP/mCherry ratio in the Δ*ncs2* mutant, but not in the wild-type strain (Figure 5E and Supplementary Figure S7B). This result demonstrates that tRNA thiolation specifically enhances the expression of genes rich in codons read by thiolated tRNAs.

### tRNA thiolation regulates *M. oryzae* virulence partially by promoting the translation of Rpd3

**Figure 6.**
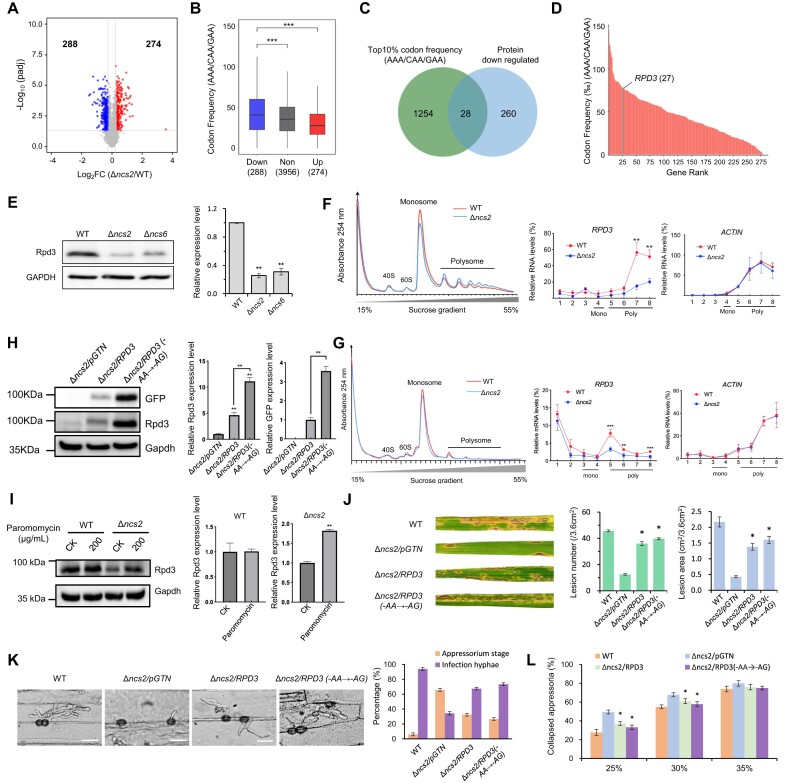
The absence of tRNA thiolation resulted in decreased levels of protein rich in AAA/CAA/GAA codons. (**A**) Volcano plot of differentially detected proteins in Δ*ncs2* compared to wild-type. Right points, log_2_ foldchange ≥ 0.263; left points, log_2_ foldchange≤-0.263; middle points, not significant. *P*< 0.05 (two-tailed *t*-test). n = 3 samples per group. (**B**) Comparison of AAA/CAA/GAA codons frequency between downregulated proteins versus upregulated and non-change proteins. Statistics calculated by Wilcoxon test. ***P< 0.001. (**C**) Venn diagram of the genes with high frequency (top 10%) in AAA/CAA/GAA codons and decreased protein level (≤1.2-fold). (**D**) Codon frequency ranking of AAA/CAA/GAA among downregulated proteins. (**E**) Western blot for Rpd3 protein level detection using the specific antibody anti-Rpd3. Anti-GAPDH antibody was used as control. The expression levels were estimated by calculating the signal intensity of bands. Statistics calculated by Student’s *t*-test. **P< 0.01. (**F**) Polysome profiling of the mycelia samples of wild-type and Δ*ncs2* mutant, and the distribution of *RPD3* transcripts in sucrose gradient fractions. *ACTIN* was used as a control. Mono, monosome; Poly, polysome. Statistics calculated by Student’s *t*-test. **P< 0.01. (**G**) Polysome profiling of the appressoria samples of wild-type and Δ*ncs2* mutant, and the distribution of *RPD3* transcripts in sucrose gradient fractions. *ACTIN* was used as a control. Mono, monosome; Poly, polysome. Statistics calculated by Student’s *t*-test. **P< 0.01. (**H**) Western blot analysis of the Rpd3 protein levels in Δncs2 strains with overexpressed Rpd3, using Anti-GFP and Anti-Rpd3 antibodies. Δ*ncs2*/RPD3 expressed wild-type RPD3 mRNA, while Δ*ncs2*/RPD3(-AA→-AG) expressed codon-optimized RPD3 mRNA. Anti-GAPDH served as a loading control. Expression levels were quantified by band intensity. Statistical significance was determined by Student’s *t*-test, with *P*< 0.01 indicating significance. (**I**) Western blot analysis of the Rpd3 protein level in mycelia samples of wild-type and Δ*ncs2* mutant under paromomycin treatment (200 μg/ml). (**J**) Virulence test of the Δ*ncs2* mutant overexpressing wild-type *RPD3* and recoded *RPD3* (-AA→-AG), and the lesion number and total lesion area was measured. Empty vector pGTN was introduced into Δ*ncs2* as control. The data represent mean values with standard deviations of three biological replicates. * denotes significant differences compared to Δ*ncs2*/pGTN (*P*< 0.01, Student’s *t*-test, two-tailed). (**K**) Penetration observation on barley epidermises at 24 hpi for the Δ*ncs2* mutant overexpressing wild-type *RPD3* and recoded *RPD3* (-AA→-AG), under a microscope. Bar = 20 μm. The data represent mean values with standard deviations of three biological replicates, and at least 50 appressoria were counted for each time. (**L**) Cytorrhysis assay for the Δ*ncs2* mutant overexpressing *RPD3* wild-type *RPD3* and recoded *RPD3* (-AA→-AG). The appressorium turgor pressure of the strains was detected using PEG8000 solutions. Percentages of the collapsed appressoria were calculated under a microscope. The data represent mean values with standard deviations of three biological replicates, and at least 50 appressoria were counted for each time. * denotes significant differences compared to Δ*ncs2*/pGTN (*P*< 0.05, Student’s *t*-test, two-tailed).

To further examine the effect of tRNA thiolation on protein production, we compared protein levels between wild-type and Δ*ncs2* strains by using quantitative mass spectrometry. Deleting NCS2 led to increased levels of 274 proteins and decreased levels of 288 proteins (Figure 6A and Supplementary Table S5). Consistent with Ribo-seq data, both GO and KEGG analyses on proteome data revealed that amino acid metabolism pathways are upregulated upon *NCS2* deletion (Supplementary Figure S8A and B). Importantly, we found that the frequency of CAA/AAA/GAA codons negatively correlates with the effect of *NCS2* deletion on protein levels (Figure 6B). In contrast, the frequency of CAG/AAG/GAG codons was not associated with protein level changes (Supplementary Figure S9). These data strongly suggest that tRNA thiolation affects protein production in a codon frequency-related manner in *M. oryzae*.

Based on these findings, we hypothesized that tRNA thiolation regulates *M. oryzae* pathogenicity by affecting the translation of proteins enriched in CAA/AAA/GAA codons. To identify these proteins, we ranked all *M. oryzae* protein-coding genes based on their CAA/AAA/GAA codon frequency. Among the top 10% of genes with the highest CAA/AAA/GAA codon frequency, only 28 genes exhibited reduced protein levels as revealed by mass spectrometry (Figure 6C and Supplementary Table S6). Rpd3, a Class I histone deacetylase, is among these 28 proteins. Moreover, the CAA/AAA/GAA codon frequency of Rpd3 ranked 27th among all the 288 genes with reduced protein levels in the Δ*ncs2* mutant (Figure 6D). Importantly, previous studies have shown that Rpd3 regulates the development and pathogenicity in *M. oryzae* (57,58), and is essential for glycogen degradation during the appressorium maturation process. As observed in the Δ*ncs2* and Δ*ncs6* mutant, knockdown of *RPD3* resulted in increased rapamycin sensitivity, abnormal autophagy, delayed glycogen utilization and impaired appressorium penetration (57,59). Therefore, we hypothesized that tRNA thiolation regulates *M. oryzae* pathogenicity by affecting the translation of Rpd3.

To test this hypothesis, we first compared Rpd3 protein levels in hyphae between wild-type and thiolation-defective strains using specific antibodies recognizing endogenous Rpd3. We found that Rpd3 protein levels in the Δ*ncs2* and Δ*ncs6* mutant were about 20% of those in the wild-type strain (Figure 6E). Moreover, *RPD3* mRNA levels associated with translating ribosome were lower in the Δ*ncs2* mutant than in wild-type mycelia cells (Figure 6F), indicating that loss of tRNA thiolation results in decreased translation of *RPD3* and consequently reduced protein level. In developing appressoria, less *RPD3* mRNAs were engaged active translation in the Δ*ncs2* mutant than in wild-type appressoria (Figure 6G), leading to reduced Rpd3 protein levels, consistent with the observation in mycelia (Supplementary Figure S10A). Collectively, these results indicated thar tRNA thiolation is critical for maintaining Rpd3 translation in both appressoria and mycelia.

To confirm that the loss of *PRD3* mRNA translation was due to inefficient decoding of CAA/AAA/GAA codons following the loss of tRNA thiolation, we recoded Rpd3 by replacing all AA-ending codons with synonymous AG-ending codons (which do not require the s^2^U_34_ modification for decoding). As expected, more Rpd3 proteins were generated from the recoded *RPD3* mRNAs than the original version (Figure 6H; Supplementary Figure S10B). To further confirm this conclusion, we treated the cells with paromomycin, which alleviates ribosomal pausing at AA-ending codons in strains lacking tRNA U_34_ thiolation modification by enhancing the acceptance of near-cognate tRNAs both in yeast and *M. oryzae* (29,39). Paromomycin treatment resulted in increased Rpd3 protein levels in the Δ*ncs2* mutant compared to wild-type cells (Figure 6I). Together, these results demonstrate that tRNA thiolation is essential for the optimal expression of Rpd3 by enhancing the decoding of AAA/CAA/GAA codons. Importantly, we found that overexpression of the original Rpd3 in the Δ*ncs2* mutant led to a partially increase in plant infection compared to expressing an empty vector, and overexpression of the recoded Rpd3 (-AA→-AG) resulted in a further increase in plant infection (Figure 6J). Correspondingly, overexpression of both the original and recoded Rpd3 (-AA→-AG) also partially ameliorated the defects in penetration and turgor pressure observed in the Δ*ncs2* mutant (Figure 6K and L). Collectively, these findings demonstrate that tRNA thiolation influences *M. oryzae* virulence partially by enhancing the translation of Rpd3, a protein enriched in AAA/CAA/GAA codons.

### tRNA thiolation also affects the translation of proteins critical for appressorium function

**Figure 7:**
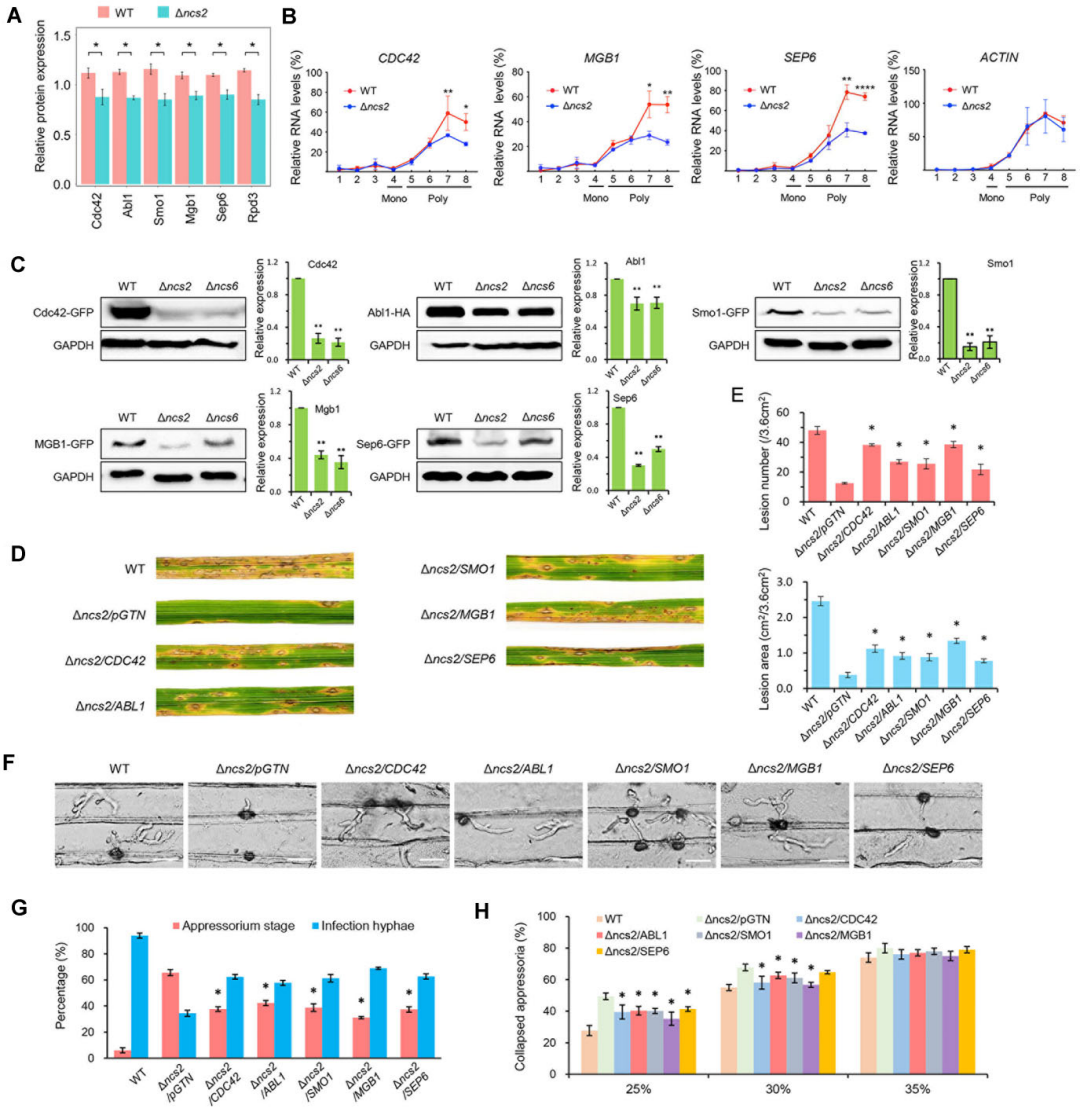
tRNA thiolation affects the pathogenicity via the protein levels of appressorium-related proteins. (**A**) Relative protein expression level of Cdc42, Abl1, Smo1, Mgb1 and Sep6 in the wild type and Δ*ncs2*. *P< 0.05. (**B**) Polysome-qPCR analysis of *CDC42*, *MGB1* and *SEP6* in the wild-type and Δ*ncs2* mutant. Actin was used as control. Mono, monosome; Poly, polysome. Statistics calculated by Student’s *t*-test. **P< 0.01. (**C**) Western blot for protein level detection. HA-Tag (for Abl1) or GFP-Tag (for Cdc42, Smo1, Mgb1 and Sep6) fusion expression vectors were transformed into the wild-type, Δ*ncs2* and Δ*ncs6* strains, and the proteins were detected by anti-HA antibody or anti-GFP antibody, respectively. Anti-GAPDH antibody was used for the control. The expression levels were estimated by calculating the signal intensity of bands. Statistics calculated by Student’s *t*-test. **P< 0.01. WT, wild type; Δ*ncs2*, *NCS2* knock-out mutant; Δ*ncs6*, *NCS6* knock-out mutant. (**D**) Virulence test of the Δ*ncs2* mutant overexpressing *CDC42*, *ABL1*, *SMO1*, *MGB1* or *SEP6*, Empty vector pGTN was introduced into Δ*ncs2* as control. (**E**) The statistics of lesion number and total lesion area. The data represent mean values with standard deviations of three biological replicates. * denotes significant differences compared to Δ*ncs2*/pGTN (*P*< 0.05, Student’s *t*-test, two-tailed). (**F**) Penetration observation on barley epidermises at 24 hpi for strains in panel (D) under a microscope. Bar = 20 μm. (**G**) Statistics for the percentages of appressoria with infection hyphae. The data represent mean values with standard deviations of three biological replicates, and at least 50 appressoria were counted for each time. * denotes significant differences compared to Δ*ncs2*/pGTN (*P*< 0.05, Student’s *t*-test, two-tailed). (**H**) Cytorrhysis assay for strains in panel (D) using PEG8000 solutions. Percentages of the collapsed appressoria were calculated under a microscope. The data represent mean values with standard deviations of three biological replicates, and at least 50 appressoria were counted for each time. * denotes significant differences compared to Δ*ncs2*/pGTN (*P*< 0.05, Student’s *t*-test, two-tailed).

Because overexpression Rpd3 only partially rescued the defects caused by Ncs2 deletion, we reasoned that tRNA thiolation may regulates *M. oryzae* virulence by affecting the expression of other proteins critical for appressorium function. Indeed, we found that the protein levels of several key proteins, including Rho-family small GTP-binding protein Cdc42 (60), AMPK β subunit-like protein Abl1 (61), GTPase-activating protein Smo1 (62), G-protein beta subunit Mgb1 (63) and septin guanosine triphosphatase Sep6 (7), were significantly reduced upon Ncs2 deletion (Figure 7A). Moreover, the translation of *CDC42*, *MGB1* and *SEP6* mRNAs was also significantly reduced in the Δ*ncs2* mutant (Figure 7B), similar to what was observed for *RPD3* mRNA. Consistently, in the Δ*ncs2* and Δ*ncs6* strains, protein levels of GFP-tagged Cdc42, Abl1, Smo1, Mgb1 and Sep6, driven by their native promoters, were significantly lower compared to the wild-type strain (Figure 7C). In contrast to the protein level changes, the transcription level of these key proteins did not show significant change between wild-type and Δ*ncs2* mutant strains (Supplementary Figure S11). However, the frequency of CAA/AAA/GAA codons in these proteins are lower than that of Rpd3, suggesting that tRNA thiolation may regulate the expression of these appressorium-related proteins through both direct and indirect mechanisms.

Importantly, overexpression of *CDC42*, *ABL1*, *SMO1*, *MGB1* and *SEP6* in the Δ*ncs2* mutant partially rescued the defects in virulence, penetration and turgor pressure observed in the Δ*ncs2* mutant (Figure 7D–H). These results collectively demonstrate that tRNA thiolation supports the virulence and appressoria functionality of *M. oryzae* by influence the translation of key proteins, such as Cdc42, Abl1, Smo1, Mgb1, Sep6 and Rpd3.

## Discussion

**Figure 8.**
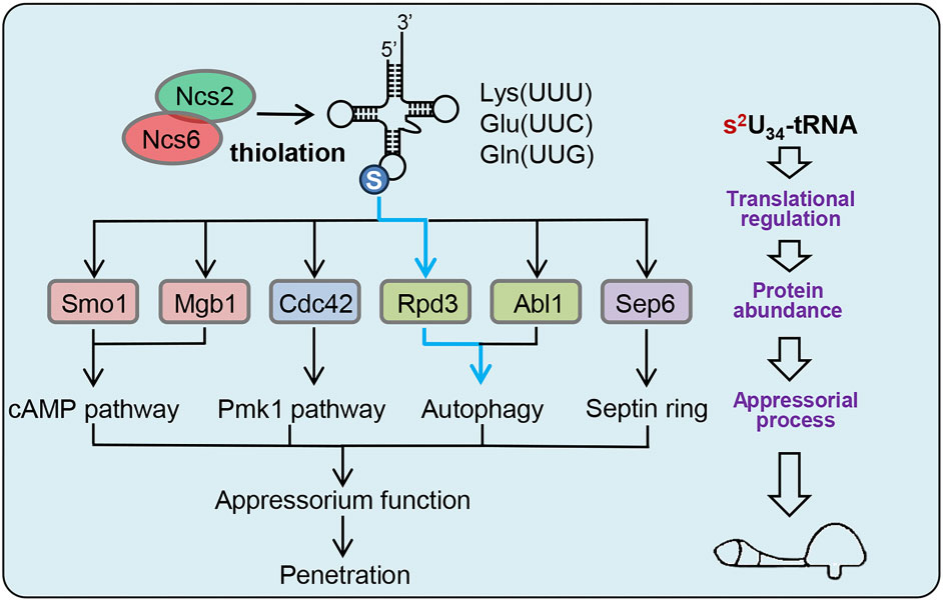
Working model for tRNA thiolation supporting pathogenicity of *M. oryzae*. The Ncs2/Ncs6 complex mediates the thiolation modification on the U_34_ base of tK^UUU^, tQ^UUG^ and tE^UUC^, which coordinates the translation efficiency of the cognate codons-rich proteins such as Rpd3, Cdc42, Abl1, Smo1, Mgb1 and Sep6. These proteins play key roles in appressorium function through cAMP signal pathway, Pmk1 signal pathway, autophagy and septin ring formation, which ultimately impacts the penetration and virulence of *M. oryzae*.

The proper functioning of the appressorium is essential for successful plant infection by *M. oryzae*. In this study, we demonstrate that, beyond its role in cytoplasmic effector secretion, tRNA s^2^U_34_ modification is critical for the full virulence of *M. oryzae* by supporting appressorium functionality. We propose a working model to illustrate the role of tRNA thiolation in appressorium-mediated plant infection by *M. oryzae* (Figure 8). In this model, Ncs2/Ncs6 collaborate with Urm1 and Uba4 to mediate thiolation in three specific tRNAs: tK^UUU^, tQ^UUG^ and tE^UUC^. The absence of this modification causes ribosome pausing on cognate codons, lading to a reduced abundance of proteins enriched in these codons, such as Cdc42, Abl1, Smo1, Mgb1, Sep6 and Rpd3. These proteins are crucial for the development and maturation of appressorium through various pathways, including the cAMP signal pathway, Pmk1 signal pathway, autophagy and septin ring formation. Ultimately, these deficiencies reduce the pathogenicity of *M. oryzae*.

A key study has highlighted the necessity of tRNA thiolation for the functioning of secreted cytoplasmic effectors in *M. oryzae* (39). The study found that without Urm1 and Uba4, essential for the s^2^U_34_ modification pathway, the secretion of effectors into the BIC is impeded, affecting growth within the host (39). This discovery has been a significant leap in our understanding of tRNA thiolation’s role in fungal pathogenesis. Expanding on this, our research investigates the impact of tRNA modification and codon usage on the appressoria function in *M. oryzae*. We have found that the s^2^U_34_ modification is crucial for appressorium maturation and penetration, a key process in fungal infection. By combining our findings with those of Li*et al.*, we offer a broader perspective on the impact of tRNA thiolation on *M. oryzae*’s pathogenicity. Our study underscores tRNA thiolation’s importance in fungal virulence and reveals its significant influence on infection mediated by appressoria.

This study aligns with previous research in *S. cerevisiae*, *C. elegans* and *C. albicans*, where the loss of tRNA thiolation also leads to increased codon-specific ribosome pausing (29,35). This suggests a conserved function for the s^2^U_34_ modification. In *M. oryzae*, the lack of tRNA thiolation disrupts translation at codons decoded by thiolated tRNAs, verified by our reporter assay (Figure 5E). Consequently, this reduction in translation impacts proteins such as Cdc42, Abl1, Smo1, Mgb1, Sep6 and Rpd3. Notably, both Smo1 and Mgb1 are involved in cAMP signal pathway, which take part in the cytoskeleton organization at the appressorium pore (62,63). Cdc42 is involved in activating the Pmk1 cascade, which is required for turgor establishment and cell polarity (60). Both Abl1 and Rpd3 are associated with the TOR kinase pathway and autophagy, which is necessary for glycogen mobilization and degradation (57,61). Sep6 is one of the septin guanosine triphosphatases that scaffold, assemble and polymerize F-actin network into the septin ring which is necessary for penetration peg formation (7). The diminished levels of these proteins in the Δ*ncs2* mutant account for its observed phenotypes, such as abnormal autophagy, impaired glycogen degradation, inadequate turgor establishment, flawed septin ring formation and unsuccessful penetration. Further supporting this, the introduction of extra copies of these genes into the Δ*ncs2* mutant partially rescued these defects, affirming the causal relationship between the lack of tRNA thiolation and these functional impairments in the pathogenicity of *M. oryzae*.

The Δ*ncs2* and Δ*ncs6* mutants exhibit reduced lesion formation and slower expansion rates compared to the wild type when inoculated on wounded rice leaves. This suggests an impairment in the extension of infection hyphae, aligning with the phenotype observed in Δ*uba4* and Δ*urm1* mutants. Microscopic examination revealed that Δ*ncs2* and Δ*ncs6* mutants also show altered hyphal growth and extension during the early stages of infection. The mutants’ hyphae are less capable of extending between cells, which hinders the formation of effective infection structures and impacts lesion development. The loss of tRNA thiolation modification in the *ncs2/6* mutants may also lead to impaired translation and secretion of effector proteins, like Δ*uba4* and Δ*urm1* mutants, subsequently affecting the extension of infection hyphae and lesion formation. Urm1 and Uba4 are involved not only in tRNA thiolation but also in protein urmylation, which has been shown is critical for stress response in yeast by driving protective phase separation of functionally critical proteins. Therefore, it is also possible that Urm1 and Uba4 may affect *M. oryzae* infection by contacting various stress presented by the host through protein urmylation other than RNA thiolation (Supplementary Figure S12) (51,64,65).

In addition to the thiolation at the 2-position of U_34_ (s^2^U_34_), another modification, mcm^5^, occurs at the 5-position (mcm^5^U_34_). These s^2^ and mcm^5^ modifications are often found together as mcm^5^s^2^U_34_, though they can also exist independently (20,34). The sulfur at the 2-position endows mcm^5^s^2^U_34_ with conformational rigidity, largely fixing the C3’-endo ribose puckering and thereby ensuring stable and accurate codon–anticodon pairing (20). Similar to the s^2^ modification, the mcm^5^ modification enhances the decoding efficiency of AAA/CAA/GAA codons (66). Intriguingly, the simultaneous absence of both modifications proves lethal in certain yeast strains (16,29). Elp3, a catalytic subunit of the Elongator complex, is responsible for the mcm^5^ modification both in yeast and *Aspergillus fumigatus* (Supplementary Figure S12) (65,67,68). In *M. oryzae*, the deletion of Elp3’s homolog results in defects in vegetative growth, conidiation, and turgor accumulation during appressoria development, mirroring the phenotypes we observed with *NCS2* and *NCS6* deletion. However, certain phenotypes reported in the *ELP3* deletion mutant, such as hyper-activated autophagy and increased sensitivity to environmental stresses [NaCl, Congo red (CR) and Calcofluor white (CFW)], were not observed in the *NCS2* and *NCS6* deletion mutants in our study (69). This could imply distinct roles for s^2^ and mcm^5^ modifications in *M. oryzae*, or alternatively, Elp3 might exert additional effects through its histone acetyltransferase activity.

In summary, our study underscores the crucial role of Ncs2/Ncs6-mediated tRNA thiolation in regulating appressorium function, contributing to a deeper understanding of *M. oryzae* pathogenicity and offering broader insights into the role of tRNA modifications in plant-pathogenic fungi. Additionally, our recent findings indicate that tRNA m^1^A methylation is essential for appressorium-mediated plant infection by regulating ergosterol biosynthesis in *M. oryzae* (70). Likewise, APH1-mediated tRNA m^7^G modification is critical for host invasion by appressoria in the phytopathogenic fungus *Colletotrichum lagenarium* (71). These findings raise the intriguing possibility that tRNA modification-mediated translational control is a conserved mechanism across fungal pathogens for maintaining appressorium function and enabling successful plant infection.

## Supplementary Material

gkae1302_Supplemental_Files

## Data Availability

The data underlying this study are available within the paper. All strains, oligonucleotides and plasmids used in this study are listed in Supplementary Table S2 and S3. The description of the algorithms applied are found in the methods section. Ribo-seq and RNA-seq data can be obtained from the China National Center for Bioinformation database with accession number PRJCA025797. Proteomics data have been deposited in the iProX database with accession number PXD051357.
