# Supplementary material for: tRNA thiolation optimizes appressorium-mediated infection by enhancing codon-specific translation in Magnaporthe oryzae
Source: Nucleic Acids Res. 2025 Jan 7;53(1):gkae1302. doi: 10.1093/nar/gkae1302 (PMC11705076; doi:10.1093/nar/gkae1302)
Supplement: gkae1302_Supplemental_Files [file gkae1302_supplemental_files.zip › Supplementary data.pdf]

**Supplementary Information for**

# tRNA thiolation controls appressorium-mediated infection by enhancing codon-specific translation in *Magnaporthe oryzae*

Xinrong Zhang[1,2,†], Rongrong He[1,3,†], Yinan Li[1,3,†], Shuchao Ren[1,3,†], Shikun Xiang[1,2], Jing Zheng[1,2], Zhiguang Qu[1,2], Shu Zhou[1,4], Zhipeng Zhou[1,3*], Xiao-Lin Chen[1,2*]

[1] National Key Laboratory of Agricultural Microbiology, Huazhong Agricultural University, Wuhan 430070, China

[2] Provincial Key Laboratory of Plant Pathology of Hubei Province, College of Plant Science and Technology, Huazhong Agricultural University, Wuhan 430070, China

[3] College of Life Science and Technology, Huazhong Agricultural University, Wuhan 430070, China

[*] To whom correspondence should be addressed. Tel: +86 136 6712 7102; Fax: +86 027 87282130; Email: chenxiaolin@hzau.edu.cn

Correspondence may also be addressed to Zhipeng Zhou. Tel: +86 189 7107 0108; Fax: +86 027 87282130; Email: zhouzhipeng9@126.com

[†] The first four authors should be regarded as Joint First Authors.

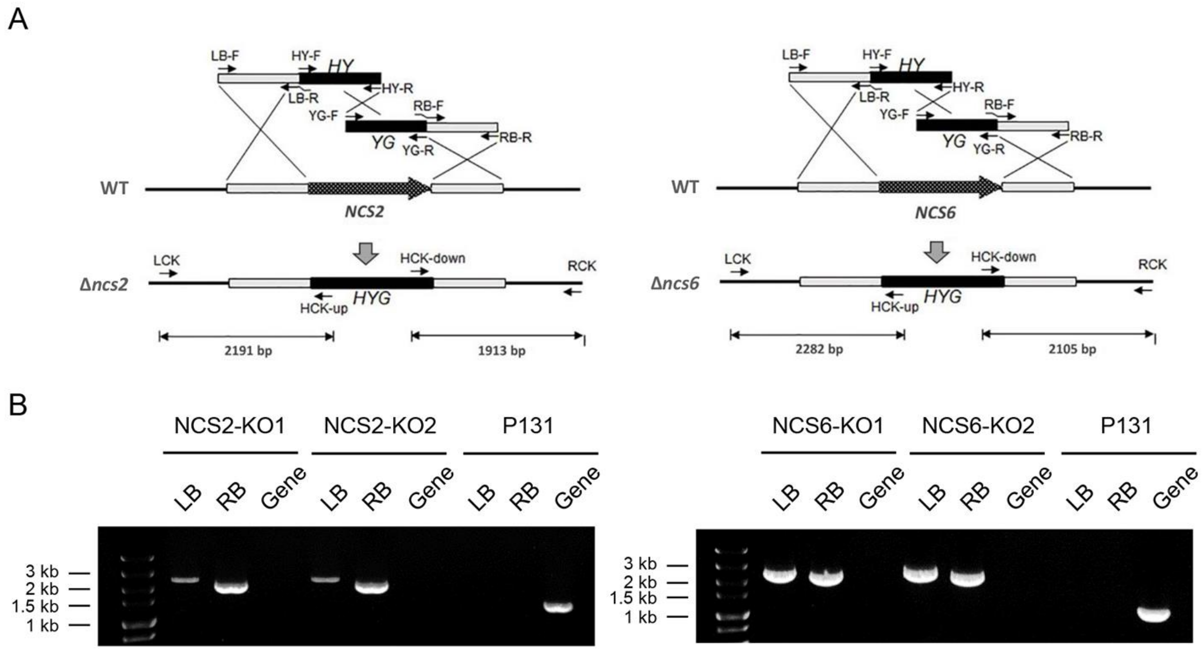


**Figure S1.** Deletion of *NCS2* and *NCS6*. (**A**) The diagram of the deletion strategy of *NCS2* and *NCS6*. (**B**) The identification of the Δ*ncs2* and Δ*ncs6* mutants by PCR. KO1 and KO2 are two independent strains.

**A**

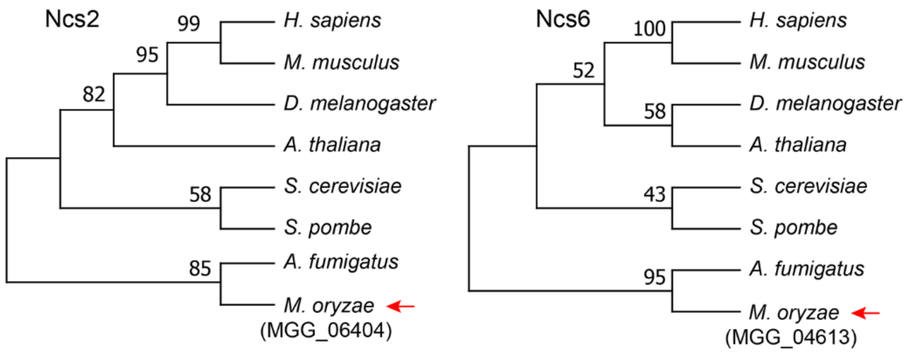


**B**

Ncs2

| Species | identity | similarity |
|---|---|---|
| *H. sapiens* | 25% | 41% |
| *M. musculus* | 26% | 40% |
| *D. melanogaster* | 22% | 40% |
| *A. thaliana* | 24% | 39% |
| *A. fumigatus* | 27% | 44% |
| *S. pombe* | 22% | 40% |
| *S. cerevisiae* | 18% | 39% |

Ncs6

| Species | identity | similarity |
|---|---|---|
| *H. sapiens* | 49% | 64% |
| *M. musculus* | 52% | 67% |
| *D. melanogaster* | 67% | 80% |
| *A. thaliana* | 64% | 79% |
| *A. fumigatus* | 71% | 79% |
| *S. pombe* | 68% | 79% |
| *S. cerevisiae* | 66% | 82% |

**Figure S2.** Phylogenetic tree and amino acid sequence identity of Ncs2 and Ncs6. (**A**) Phylogenetic tree of Ncs2 and Ncs6 proteins across Eukarya. The phylogenetic tree was constructed using MegaX software. (**B**) Protein sequence identity and similarity among homologs of Ncs2 and Ncs6.

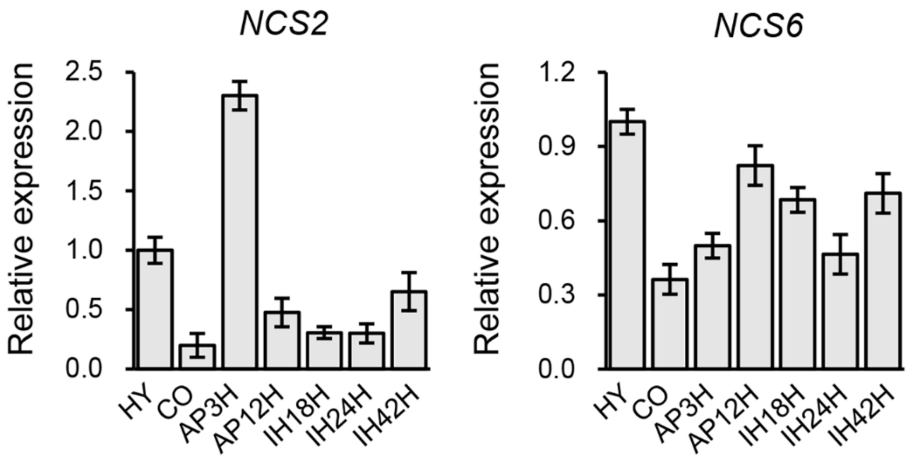


**Figure S3.** Expression patterns of *NCS2* and *NCS6* at different developmental stages. The tubulin gene was used as an internal control. Expression level of *NCS2* or *NCS6* in HY was set as 1. HY, vegetative hyphae; CO, conidia; AP3H, appressoria at 3 hpi; AP12H, appressoria at 12 hpi; IH18H, invasive hyphae at 18 hpi; IH24H, invasive hyphae at 24 hpi; IH42H, invasive hyphae at 42 hpi.

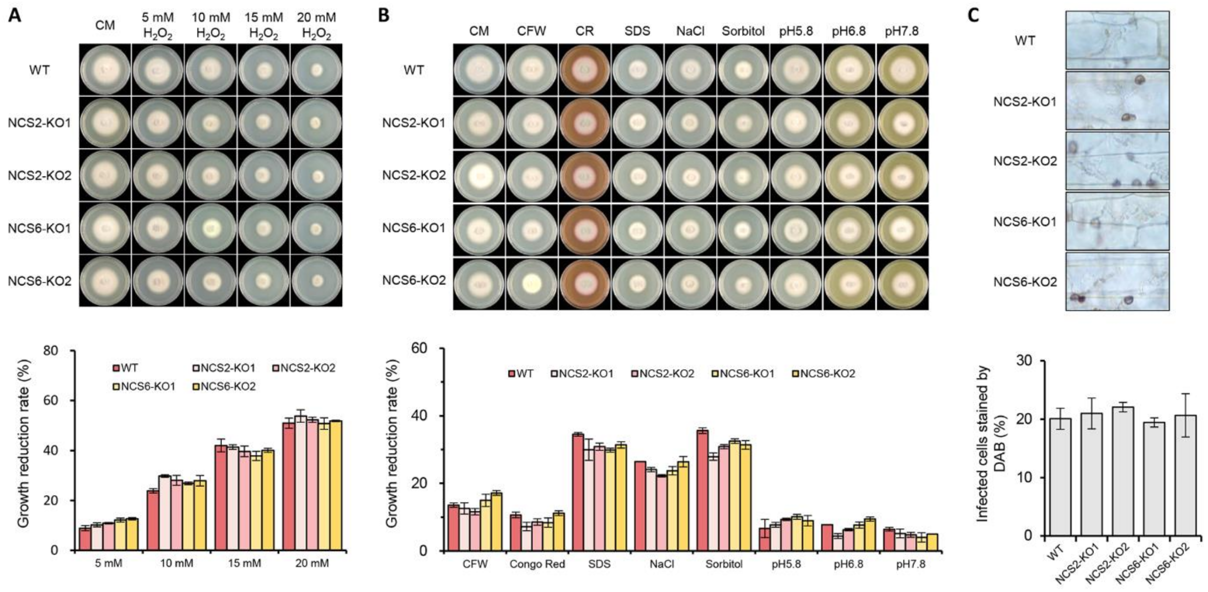


**Figure S4.** (**A**) Assay for $H_2O_2$ sensitivity. Strains were cultured on CM plates containing 5 mM, 10 mM, 15 mM and 20 mM $H_2O_2$ at 28 °C in dark for 5 days. The growth reduction rates were calculated by colony diameter. (**B**) Assay for sensitivity to different stress. Strains were cultured on CM plates containing cell wall perturbing reagents [Calcofluor white (CFW), Congo red (CR), and sodium dodecyl sulfate (SDS)], high osmotic pressure (0.5 M NaCl and 1 M sorbitol), or different pH conditions (pH 5.8, pH 6.8 and pH 7.8) at 28 °C for 5 days. The growth reduction rates were calculated by colony diameter. (**C**) DAB staining observation. Barley epidermis cells were inoculated with different strains and stained with DAB at 30 hpi for 8 h, and then observed after decolorization under a light microscopy. Bar = 20 μm. Percentages of infected cells which stained by DAB was calculated. The data represent mean values with standard deviations of three biological replicates. WT, wild type; NCS2-KO1 and NCS2-KO2, two independent Δ*ncs2* mutants; NCS6-KO1 and NCS6-KO2, two independent Δ*ncs6* mutants.

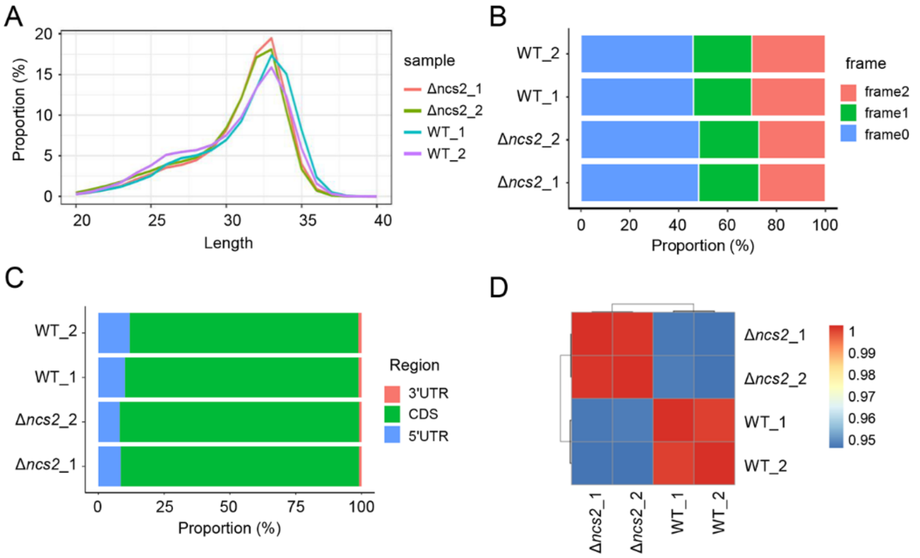


**Figure S5.** Landscape of Ribo-seq data at codon resolution. (**A**) RPFs length distribution of each sample. (**B**) Proportion of RPFs mapped to each of three reading frames. Frame 0 corresponds to the correct reading frame of each transcript. (**C**) Proportion of RPFs mapped to 5′ UTR, CDS, and 3′ UTR for each sample. (**D**) Correlation of gene expression between each sample.

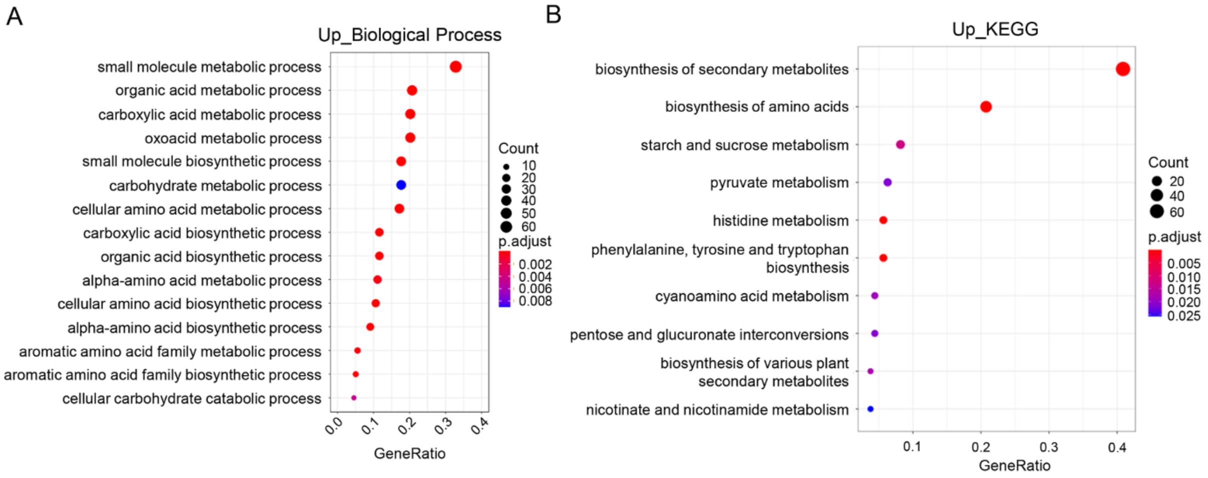


**Figure S6.** GO and KEGG pathway enrichment analysis of up-regulated RPFs in the Δ*ncs2* mutant.

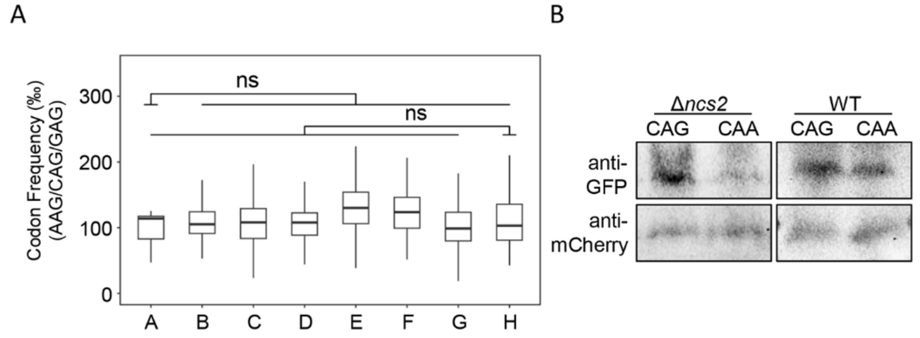


**Figure S7.** Comparison of AAG/CAG/GAG codons frequency and western blot analysis for the reporter assay. (**A**) Comparison of AAG/CAG/GAG codons frequency between genes through classification based on fold changes of RPFs and mRNAs. Statistics calculated by student t-test. *$p < 0.05$. (**B**) Western blot analysis for the reporter assay. The amounts of GFP proteins were detected by anti-GFP antibody. Anti-mCherry antibody was used as control.

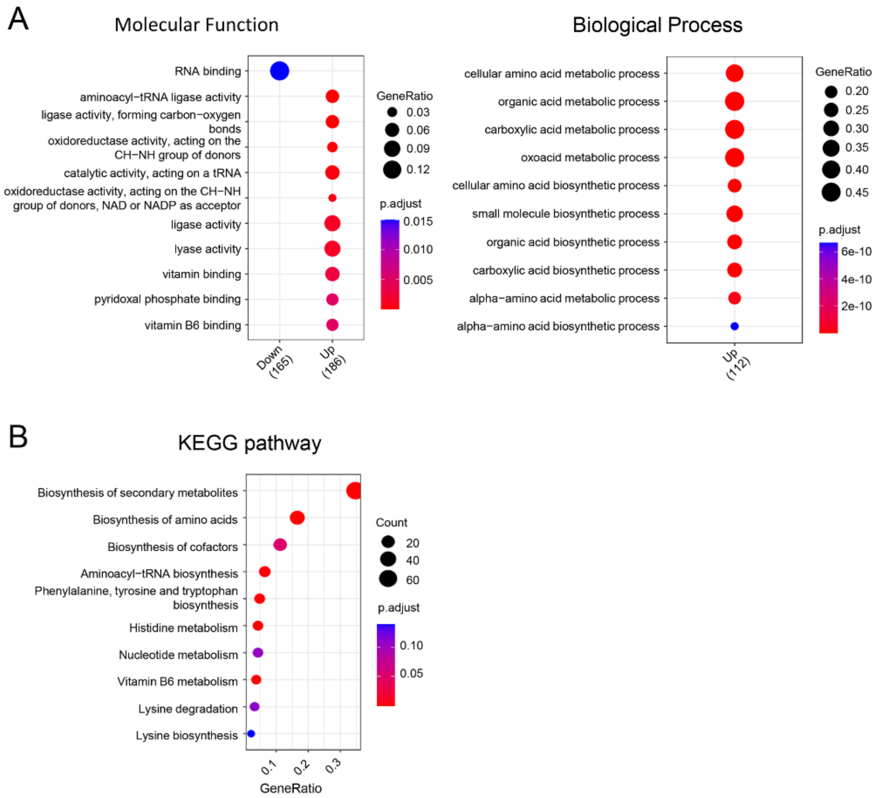


**Figure S8.** GO and KEGG pathway enrichment analysis of up- or down-regulated proteins in the Δ*ncs2* mutant.

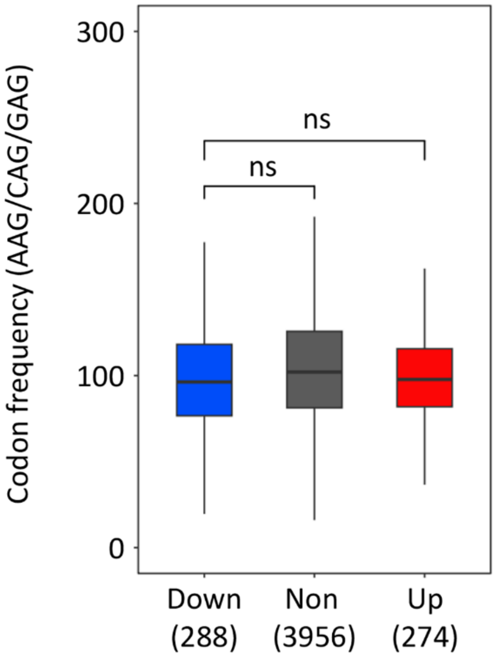


**Figure S9.** Comparison of synonymous codons frequency between down-regulated proteins versus up-regulated and non-change proteins. Statistics calculated by Wilcoxon test. ns, not significant.

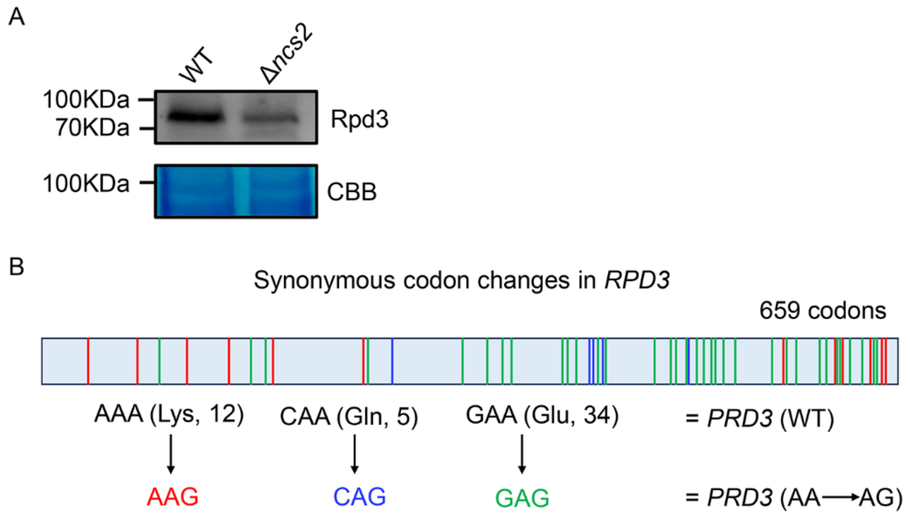


**Figure S10.** Rpd3 protein levels in WT and Δ*ncs2* appressoria samples and distribution of AA-ending and AG-ending condos in *RPD3*. (**A**) Comparison of Rpd3 protein level in WT and Δ*ncs2* appressoria samples. (**B**) Distribution of AA-ending and AG-ending condos in *RPD3*.

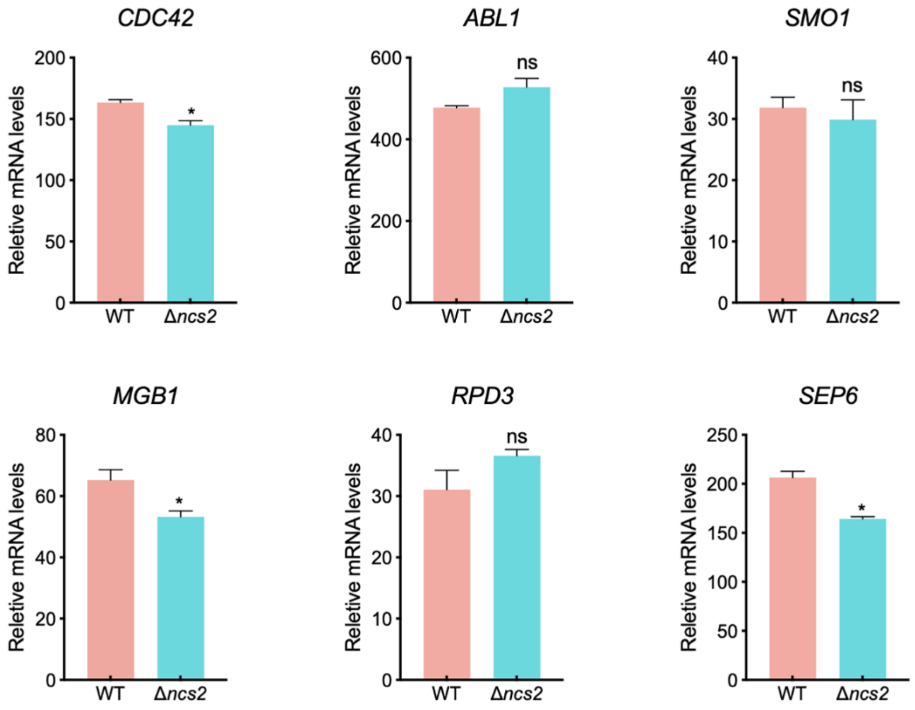


**Figure S11.** Comparison of *CDC42*, *ABL1*, *SMO1*, *MGB1*, *RPD3*, and *SEP6* transcription levels in WT and Δ*ncs2* mutant strain based on RNA-seq.

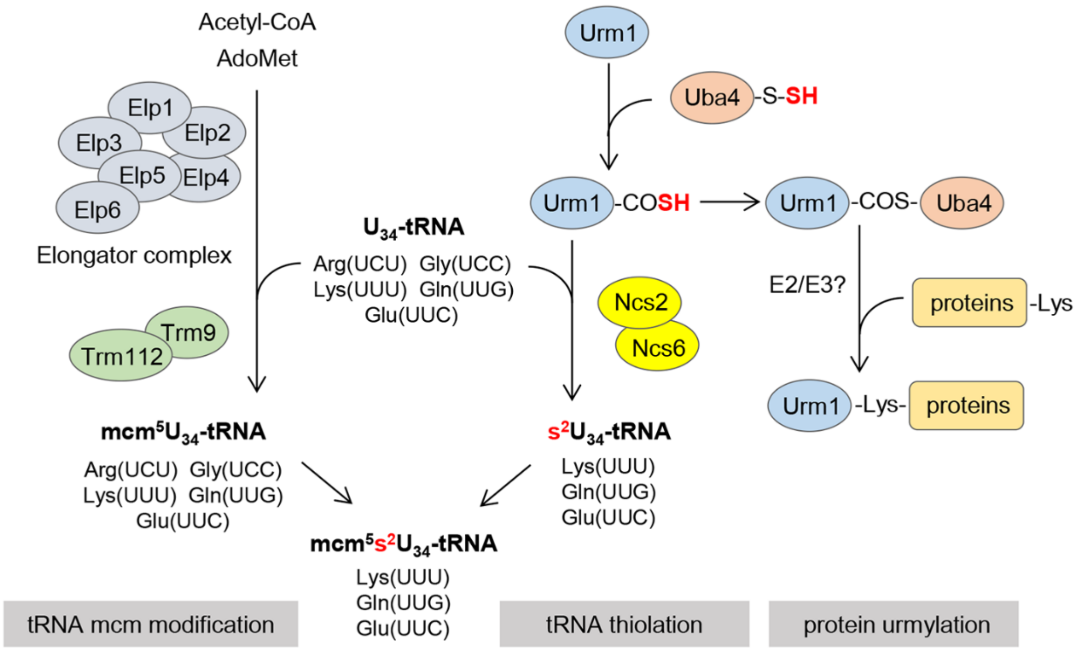


**Figure S12.** Pathways of tRNA wobble uridine $U_{34}$ modification. Urm1 and Uba4 integrate tRNA thiolation and protein urmylation. For tRNA thiolation, Urm1 and Uba4 act as sulfur donors, transfering the sulfur to $tK^{UUU}$, $tQ^{UUG}$ and $tE^{UUC}$ via the Ncs2/Ncs6 complex to generate $s^2U_{34}$-tRNA. $U_{34}$-tRNA can also be modified by $mcm^5$ side chain through Elp1-6 complex and Trm9/Trm112 complex. The $s^2$ modification and $mcm^5$ modification can exist either separately or together, which called $mcm^5s^2U$ modification.

**Table S1.** Fungal strains used in this study.

| Strains | Discriptions |
|---|---|
| WT | P131, a wild-type isolate of *M. oryzae* |
| NCS2-KO1 | One independent *NCS2* deletion mutant of P131 |
| NCS2-KO2 | Another independent *NCS2* deletion mutant of P131 |
| NCS6-KO1 | One independent *NCS6* deletion mutant of P131 |
| NCS6-KO2 | Another independent *NCS6* deletion mutant of P131 |
| NCS2com | Complementary strain of NCS2-KO1 |
| NCS6com | Complementary strain of NCS6-KO1 |
| NCS2:GFP | Colocalization strain of Ncs2 in P131 |
| NCS6:GFP | Colocalization strain of Ncs6 in P131 |
| NCS6:GFP+ NCS2:3×Flag | NCS6:GFP fusion protein and Ncs2:3×Flag fusion protein co-expressed in P131 strain |
| NCS2:3×Flag | P131 strain expressing Ncs2:3×Flag fusion protein |
| WT/*GFP:ATG8* | P131 strain expressing GFP:Atg8 fusion protein |
| Δ*ncs2/GFP:ATG8* | *NCS2* deletion mutant expressing GFP:Atg8 fusion protein |
| Δ*ncs6/GFP:ATG8* | *NCS6* deletion mutant expressing GFP:Atg8 fusion protein |
| WT/*SEP6-GFP* | P131 strain expressing Sep6-GFP fusion protein |
| Δ*ncs2/SEP6-GFP* | *NCS2* deletion mutant expressing Sep6-GFP fusion protein |
| Δ*ncs6/SEP6-GFP* | *NCS6* deletion mutant expressing Sep6-GFP fusion protein |
| WT/6×CAA | P131 strain expressing 6×CAA vector |
| WT/6×CAG | P131 strain expressing 6×CAG vector |
| Δ*ncs2*/6×CAA | *NCS2* deletion mutant expressing 6×CAA vector |
| Δ*ncs2*/6×CAG | *NCS2* deletion mutant expressing 6×CAG vector |
| WT/*ABL1* | P131 strain expressing Abl1-HA fusion protein |
| Δ*ncs2/ABL1* | *NCS2* deletion mutant expressing Abl1-HA fusion protein |
| Δ*ncs6/ABL1* | *NCS6* deletion mutant expressing Abl1-HA fusion protein |
| WT/*CDC42* | P131 strain expressing Abl1-HA fusion protein |
| Δ*ncs2/CDC42* | *NCS2* deletion mutant expressing Abl1-HA fusion protein |
| Δ*ncs6/CDC42* | *NCS6* deletion mutant expressing Abl1-HA fusion protein |
| WT/*MGB1* | P131 strain expressing MGB1-GFP fusion protein |
| Δ*ncs2/MGB1* | *NCS2* deletion mutant expressing MGB1-GFP fusion protein |
| Δ*ncs6/MGB1* | *NCS6* deletion mutant expressing MGB1-GFP fusion protein |
| WT/*SMO1* | P131 strain expressing Smo1-GFP fusion protein |

| | |
|---|---|
| Δ*ncs2/SMO1* | *NCS2* deletion mutant expressing Smo1-GFP fusion protein |
| Δ*ncs6/SMO1* | *NCS6* deletion mutant expressing Smo1-GFP fusion protein |
| Δ*ncs2/RPD3* | *NCS2* deletion mutant expressing Rpd3-GFP fusion protein |
| Δncs2/RPD3(-AA→-AG) | NCS2 deletion mutant expressing recoded Rpd3-GFP fusion protein where all AA-ending codons were recoded to AG-ending codons in *RPD3* |
| Δ*ncs2/pGTN* | Empty vector pGTN was introduced into *NCS2* deletion mutant |

**Table S2.** Primers used in this study.

| Primers | Sequences (5'–3') |
|---|---|
| *NCS2* gene-up | TCCAAGAAATTCGGACTGAT |
| *NCS2* gene-down | ATAGCTTGCTGCTTCCCTGT |
| *NCS6* gene-up | CGCTATGTGTCTTGTGTGGT |
| *NCS6* gene-down | ACCTGTCTGGTAACTGTTCCA |
| *NCS2* LCK | TGGCTGTAGGCGTAGATCCA |
| *NCS2* RCK | GGTCGCTACCAATTCGTTGAT |
| *NCS6* LCK | TGCAAAGGGACGGTATGACT |
| *NCS6* RCK | GACTCCTCGGCACTTTCACT |
| HCK-up | GACAGACGTCGCGGTGAGTT |
| HCK-down | TCTGGACCGATGGCTGTGTAG |
| *NCS2* GTN-F | GATAAGCTTGATATCGAATTCCAAACACTTCTGGATGTCAGCA |
| *NCS2* GTN-R | ACCTCTAGAACTAGTGGATCCCCCATTGACAGATCTCTGGC |
| *NCS6* GTN-F | GATAAGCTTGATATCGAATTCCGCCGAATGCCTACCTTCT |
| *NCS6* GTN-R | ACCTCTAGAACTAGTGGATCCATTAGTATCACCAACCTGTATCTGTGGTC |
| *NCS2* KNFlag-F | AAACCCGGGCTGCAGGAATTCATGGCGGAAATGGCCGAC |
| *NCS2* KNFlag-R | TTCGAGGTCGACGGTATCGATCCCATTGACAGATCTCTGGCAAC |
| Promoter-F | GCCCTTGCTCACCATATTGAGAGATACTCGGTGC |
| Promoter-R | ACCGTCGACCTCGAG GGTTTTGGCTATATGAATGGT |
| Circ PCR pGTN-F | CTCGAGGTCGACGGTA |
| Circ PCR pGTN-R | GGTACCCAGCTTTTGTTC |
| mCherry-F | ACAAAAGCTGGGTACCCTTGTACAGCTCGTCCA |
| mCherry-R | TAATTACCGGTATAATCATAATGGTGAGCAAGGGC |
| CAA/G-GFP-F | GAATTCATGGACTACAAGGACCATG |
| CAA-GFP-R | TCTAGATTGTTGTTGTTGTTGTTGCTTGTCATCATCATCC |
| CAG-GFP-R | TCTAGACTGCTGCTGCTGCTGCTGCTTGTCATCATCATCC |
| *ABL1* YTP105-F | GGTACCGGGCCCCCCCTCGAGCCGATGCGACTCGACATTAG |
| *ABL1* YTP105-R | CGCTCTAGAACTAGTGGATCCCTTGGAATCCTTGCTGGACA |
| *CDC42* GTN-F | GATAAGCTTGATATCGAATTCACCTGTCAAGCTGACACCCA |
| *CDC42* GTN-R | ACCTCTAGAACTAGTGGATCCAAGGATCAGGCACTTTTTGGA |
| *MGB1* GTN-F | GATAAGCTTGATATCGAATTCTTGTCGCCTGAGCATTGGT |
| *MGB1* GTN-R | ACCTCTAGAACTAGTGGATCCGTATGCCCAGATCTTGAGCTG |
| *SMO1* GTN-F | GATAAGCTTGATATCGAATTCCCAGGCGAAGGCAGTCGTAC |

| | |
|---|---|
| *SMO1* GTN-R | ACCTCTAGAACTAGTGGATCCCCAGCCCTTCTTCCGCG |
| *SEP6* GTN-F | GATAAGCTTGATATCGAATTCAAGCGGCGCATTTCAAGCTA |
| *SEP6* GTN-R | ACCTCTAGAACTAGTGGATCCGCGACGGCCATGCGAAC |
| *RPD3* GTN-F | GATAAGCTTGATATCGAATTCTGGGTACGGAAATCATCCATC |
| *RPD3* GTN-R | ACCTCTAGAACTAGTGGATCCTGGCTCTGCCTCCTCTGTG |
| *NCS2* qPCR-F | CAAGAAATTCGGACTGATGCT |
| *NCS2* qPCR-R | GAACGACAAGCCGACTGTG |
| *NCS6* qPCR-F | GCGCTATGTGTCTTGTGTGG |
| *NCS6* qPCR-R | GTGAAGAGGTTGGAGCTGGT |
| *TUBULIN* qPCR-F | GGACCGTATGCAGAAGGAGA |
| *TUBULIN* qPCR-R | TGAGAATGGAACCACCGATC |
| *CDC42* qPCR-F | AAGCTGAGCAAGCAGAAGAT |
| *CDC42* qPCR-R | TGCCTCATCAAAGACATCCT |
| *MGB1* qPCR-F | ATCTGTTGCGACATCAGTTT |
| *MGB1* qPCR-R | TTCTCGTGACCGACCAAAGA |
| *SEP6* qPCR-F | CACATGCTCGATCTCATCCA |
| *SEP6* qPCR-*R* | TCCTCCTCCTCCTTGAACTT |
| *RPD3* qPCR-*F* | AGGAAAGTGGGGGAAAGGAA |
| *RPD3* qPCR-R | TTCTTTGTCACCACTCTCGG |
| *ACTIN* qPCR-F | ACCACTTTCAACTCGATCAT |
| *ACTIN* qPCR-R | ATCTCCTTCTGCATACGGTC |
| $tRNA^{Lys(TTT)}$ | 5'biotin-CGCCCCAGGCGGGGGTCGAACCCGCAGCCTTCGGATTAAAA GTCCGACGCTCTAGCCAATTG |
| $tRNA^{Gln(TTG)}$ | 5'biotin-AGGTTCCGGCGAGATTCGAACTCGCGATTCGAGGATCAAAAC CTCACGTGCTAACCACTA |
| $tRNA^{Glu(TTC)}$ | 5'biotin-CTCCGATATCGGGAATCGAACCCGAGTCGCCGCGGTGAAAA CGCGGTATGCTAACCCCTACAC |
| $tRNA^{Lys(CTT)}$ | 5'biotin-AGCCCGACGTGGGGGTCGAACCCACAGCCTTGAGATTAAGA GTCTCACGCTCTACCGATT |
| $tRNA^{Gln(CTG)}$ | 5'biotin-AGGTCCTACCCGGAATCGAACCGGGATCATGAGGATCAGAA CCTCATGTGCTAACCATTA |
| $tRNA^{Glu(CTC)}$ | 5'biotin-CTCCGATATCGGGAATCGAACCCGAGTCGCCGCGGTGAAAA CGCGGTATGCTAACCCCTACAC |

**Table S3.** Plasmids used in this study.

| Plasmids | Discriptions |
|---|---|
| pGTN | Vector used to construct vectors to express selected genes fused with eGFP at the C-terminal; with the fungal native promoter. |
| pGTN-*NCS2* | Vector for *NCS2* complementation and Ncs2 sub-cellular localization; *NCS2* gene with 1.5 kb native promoter region were cloned into vector pGTN. |
| pGTN-*NCS6* | Vector for *NCS6* complementation and Ncs6 sub-cellular localization; *NCS6* gene with 1.5 kb native promoter region were cloned into vector pGTN. |
| pKNFlag | Vector used to construct vectors to constitutively express selected genes fused with 3×Flag at the C-terminal; with the fungal constitutive promoter RP27. |
| pKNFlag-*NCS2* | Vector for co-immunoprecipitation of Ncs2 and Ncs6 protein; coding region of *NCS2* was cloned into vector pKNFlag. |
| pKNTG | Vector used to construct vectors to express selected genes fused with eGFP at the N-terminal; with the fungal native promoter. |
| pKNTG-*ATG8* | Vector for Atg8 sub-cellular localization; 2 kb native promoter, *ATG8* coding region and 0.5 kb terminator were cloned into pKNTG. |
| pGTN-*SEP6* | Vector for Sep6 sub-cellular localization, for testing the abundance of Sep6 protein and for testing the effect of Sep6 on the phenotypes of Δ*ncs2*; *SEP6* gene with 1.5 kb native promoter region were cloned into vector pGTN. |
| pmCherry-6×CAA-GFP | Vector for testing the effect of CAA codon on translation; with the fungal bidirectional promoter. |
| pmCherry-6×CAG-GFP | Vector for testing the effect of CAG codon on translation; with the fungal bidirectional promoter. |
| pYIP105 | Vector used to construct vectors to express selected genes fused with HA-tag at the C-terminal; with the fungal native promoter. |
| pYIP105-*ABL1* | Vector for testing the abundance of Abl1 protein and for testing the effect of Abl1 on the phenotypes of Δ*ncs2*; ABL1 gene with 1.5 kb native promoter region were cloned into vector pYIP105. |
| pGTN-*CDC42* | Vector for testing the abundance of Cdc42 protein and for testing the effect of Cdc42 on the phenotypes of Δ*ncs2*; *CDC42* gene with 1.5 kb native promoter region were cloned into vector pGTN. |
| pGTN-*MGB1* | Vector for testing the abundance of MGB1 protein and for testing the effect of MGB1 on the phenotypes of Δ*ncs2*; *MGB1* gene with 1.5 kb native |

| | |
|---|---|
| | promoter region were cloned into vector pGTN. |
| pGTN-*SMO1* | Vector for testing the abundance of Smo1 protein and for testing the effect of Smo1 on the phenotypes of Δ*ncs2*; *SMO1* gene with 1.5 kb native promoter region were cloned into vector pGTN. |
| pGTN-*RPD3* | Vector for testing the effect of Rpd3 on the phenotypes of Δ*ncs2*; *RPD3* gene with 1.5 kb native promoter region were cloned into vector pGTN. |
| pGTN-RPD3(-AA→-AG) | Vector for testing the effect of AA-ending cognate codons on the phenotypes of Δ*ncs2*; recoded *RPD3* gene where all AA-ending codons were recoded to AG-ending codons with 1.5 kb native promoter region were cloned into vector pGTN. |

**Table S4 (separate file).** RPF levels of all genes identified by Ribo-seq.

**Table S5 (separate file).** Differentially expressed proteins in Δ*ncs2* mutant.

**Table S6.** List of the 28 down-regulated proteins with top 10% codon frequency of AAA/CAA/GAA.

| Gene id | DiscriptionsAAA/CAA/GAA codon frequency (‰) | Protein level | Discriptions |
|---|---|---|---|
| MGG_17652 | 144 | down | gag protein |
| MGG_03885 | 129 | down | snare domain-containing protein |
| MGG_15996 | 126 | down | stress responsive a b barrel domain-containing protein |
| MGG_14609 | 120 | down | protein kinase-like domain |
| MGG_17180 | 118 | down | peptidyl-prolyl cis-trans isomerase |
| MGG_04093 | 114 | down | pe repeat family protein |
| MGG_16400 | 112 | down | af264028_1gag protein |
| MGG_00594 | 97 | down | filament-forming protein |
| MGG_06433 | 95 | down | pre-mrna-splicing factor spp2 |
| MGG_02456 | 92 | down | stress response protein nst1 |
| MGG_07479 | 88 | down | kinetoplast-associated protein kap-like protein |
| MGG_16203 | 87 | down | smc1 protein |
| MGG_05681 | 86 | down | prp18 domain-containing protein |
| MGG_04988 | 85 | down | chromosome segregation protein suda |
| MGG_02510 | 85 | down | coiled-coil domain-containing protein 25 |
| MGG_17817 | 85 | down | hypothetical protein MGG_17817 |
| MGG_04346 | 84 | down | sterol 24-c-methyltransferase |
| MGG_01596 | 84 | down | camk rad53 protein kinase |
| MGG_08838 | 84 | down | glycosylphosphatidylinositol anchor synthesis protein |
| MGG_01233 | 82 | down | methyltransferase type 11 |
| MGG_01426 | 81 | down | pre-mrna-splicing factor cef-1 |
| MGG_08871 | 80 | down | hypothetical protein MGG_08871 |

| | | | |
|---|---|---|---|
| MGG_02064 | 79 | down | hypothetical protein MGG_02064 |
| MGG_06407 | 78 | down | wd repeat protein |
| MGG_09917 | 78 | down | dna topoisomerase 1 |
| MGG_00540 | 78 | down | neurofilament protein h form h2 protein |
| MGG_05857 | 77 | down | histone deacetylase rpd3 |
| MGG_06322 | 77 | down | brix domain-containing protein |
